# Dinuclear Copper(I) Complexes Featuring Metal–Metal Interactions and a μ_2_‐Bridging Phosphane With Additional Olefinic Binding Sites

**DOI:** 10.1002/chem.71085

**Published:** 2026-05-03

**Authors:** Zhongshu Li, Daniel Himmelbauer, Jan Oswald, Michael Wörle, René Verel, Zoltán Benkő, Hansjörg Grützmacher

**Affiliations:** ^1^ LIFM, IGCME School of Chemistry Sun Yat‐Sen University Guangzhou China; ^2^ Department of Chemistry and Applied Biosciences ETH Zurich Zurich Switzerland; ^3^ Department of Inorganic and Analytical Chemistry Budapest University of Technology and Economics Budapest Hungary; ^4^ HUN‐REN‐BME Computation Driven Chemistry Research Group Budapest Hungary

## Abstract

The molecule Ptrop_3_ contains in each trop unit (trop = dibenzo[a,d]cyclohepten‐5‐yl) a rigid central seven‐membered ring with an olefinic binding site making Ptrop_3_ potentially a tetradentate ligand. Nevertheless, in mononuclear Cu^I^ complexes, [Cu(Ptrop_3_)X] [X = Cl (**2**), OTf (**3**), BF_4_ (**4’**)], this phosphane behaves mainly as a highly bulky ligand (cone angle approx. 250 °) and the interactions between Cu^I^ center and the C═C_trop_ units are remarkably weak, such as in other related Cu trop‐type complexes. The reaction of [Cu(Ptrop_3_)(OTf)] with Cu^+^ sources allows the preparation of dinuclear complexes [Cu_2_(μ_2_‐Ptrop_3_)(μ_2_‐X)]^+^ (X = Cl, OTf, TFA), which contain a μ_2_‐bridging P center and in which the interaction between the Cu^I^_2_ core and the olefins is significantly enhanced. Experimental investigations and DFT calculations indicate that the uptake of a Cu^+^ ion by the mononuclear complex [Cu(Ptrop_3_)(OTf)] (**3**) is a kinetically and thermodynamically favorable process.

## Graphical abstract

A Cu_2_P triangle captured in a tetrapodal phosphane tris(olefin) ligand, where all binding forces are weak apart from the Cu─P interaction.

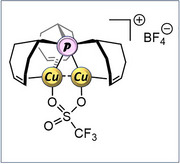

## 1 Introduction

**FIGURE 1:**
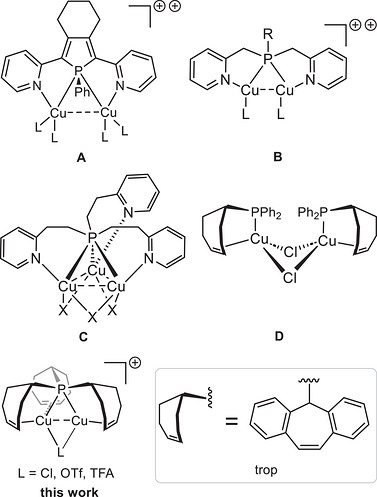
Bis and tris‐pyridyl‐substituted phosphanes serve as μ_2_‐P bridging or μ_3_‐P bridging ligands in dinuclear Cu^I^ complexes **A** and **B** and trinuclear complex **C** as ligands. Complex **D** is an early example of a Cu^I^ complex with a trop‐type olefin phosphane ligand showing a weak Cu^I^ olefin interaction.

Since the first discoveries of the μ_3_‐ and μ_2_‐bridging mode of tertiary phosphanes between three or two metal centers more than 30 years ago [1, 2], a rather wide range of complexes with μ_2_‐bridging mode of tertiary pnictogen organyl compounds ER_3_ (E = P, As, Sb) has been established [3, 4]. Among those are also complexes with a dinuclear copper core, which were first reported by Réau et al. [5, 6, 7]. In these complexes, a dinuclear copper core is bridged by a tridendate 2,5‐bis(2‐pyridyl)phosphole ligand, which bridges the two Cu^I^ centers in a μ_2_‐1κN:1,2κP:2κN fashion (see **A** in Figure 1). These compounds are remarkably stable and can be used as molecular “clips” for the selective supra‐molecular assembly of nanometer‐sized objects [8, 9]. Related complexes **B** (Figure 1) with bis[2‐pyridyl)methyl]phosphane ligands, which likewise coordinate in a μ_2_‐1κN:1,2κP:2κN fashion to a dinuclear (Cu^I^)_2_ core were reported by Klausmeyer et al. [10]. and Karaghiosoff and coworkers [11]. These complexes are interesting not only with respect to the non‐classical binding mode of the central phosphorus atom but also with respect to their distances between two d^10^‐valence configured Cu^I^ ions (referred to as d^10^‐d^10^ Cu^I^‐Cu^I^) in the rather wide range of 2.46–2.62 Å, which are designated as “cuprophilic” interactions causing sometimes special optoelectronic properties [12, 13, 14].

Recently, even a μ_3_‐bridging mode was reported for the tertiary phosphane tris[(2‐pyridyl)ethyl]phosphane, which caps a Cu_3_X_3_ core (X = Cl, Br, I). Some of these compounds show highly remarkable optoelectronic properties [15]. The bonding in the complexes was elucidated by quantum chemical methods, which indicate that the Cu_3_P interaction is best characterized as a donation of the lone‐pair at phosphorus into the 4s orbitals at the copper centers.

Noteworthy in this context is that the μ_2_‐bridging mode of trivalent phosphorus centers in neutral π‐conjugated heterocycles such as phosphines—with a λ^3^,σ^2^‐P atom as donor site—has been discovered much earlier [16]. Dinuclear copper complexes with μ_2_‐phosphinines are known through our [17, 18] and Müller's et al. work [19]. Likewise, some complexes show interesting photophysical properties. Principles for the different possible binding modes of phosphinines have been pointed out recently [20].

In all copper complexes with a μ_2_‐bridging λ^3^,σ^3^‐phosphane, the phosphorus donor center is embedded within a ligand framework that carries additional strong classical donor sites such as nitrogen in **A**–**C**. Here we report mono‐nuclear and especially dinuclear Cu^I^_2_ complexes with a μ_2_‐bridging tertiary λ^3^,σ^3^‐P center that carries only weakly electron donating olefinic units as additional potential binding sites. This is indicated by the marginally elongated coordinated C═C bonds in olefin copper complexes, which were isolated and characterized by x‐ray diffraction (XRD) methods [21]. More than 25 years ago, we reported copper complexes **D** with a chelating dibenzo[a,d]cyclohepten‐5‐yl)diphenyl phosphane (Ph_2_Ptrop) as ligand, which contains a rigid concave binding site consisting of a Ph_2_P group acting predominantly as σ‐donor and a C═C_trop_ unit as σ‐donor/ π−acceptor. However, the binding of the C═C_trop_ unit must be very weak and only a marginal lengthening of the C═C bond from 1.32 Å in the uncoordinated ligand to 1.35 Å was observed and likewise the ^1^H and ^13^C NMR shifts of the C═C_trop_ unit hardly change (a 2–3 ppm shift to higher frequencies is observed) [22]. In closely related dibenzoazepine copper complexes—where the benzylic CH group of the trop ligand is replaced by a N center rendering the central seven membered ring more flexible—even no interaction of the Cu^I^ centers with the C═C units was observed [23]. Some time ago, we reported also on P(trop)_3_ (**1**) and its Ag^I^ and Au^I^ complexes. Again, a comparison of the XRD data of **1** (Σ°(P) = 295.5 °; C═C_trop_ = 1.33 Å) and its ^1^H and ^13^NMR data in coordinated and non‐coordinated form excludes any significant interaction between the C═C_trop_ units and the coinage metal center (in contrast to complexes with late transition metal centers such as Rh^I^ or Ir^I^ where the C═C_trop_ bond length is significantly elongated to > 1.4 and sizable coordination shifts between 15 and 60 ppm to lower frequencies are observed in the ^13^C NMR data) [24]. Very recently, we found a very convenient synthesis of Ptrop_3_ from tropCl and H_3_P → AlCl_3_ and this allowed us to start to study the coordination chemistry of this highly bulky phosphane (cone angle aprox. 250 °) in more detail. Here we report the synthesis of a number of mononuclear Cu^I^ complexes and—unexpectedly—also dinuclear complexes with Ptrop_3_ as bridging μ_2_‐κ1,2‐P ligand. Moreover, we observed an equilibrium between these mononuclear and dinuclear complexes, which allowed to roughly estimate some kinetic and thermodynamic data for the conversion of a mono‐nuclear to a di‐nuclear Cu^I^ complex. DFT calculations give some insight into the electronic structure of these compounds.

## 2 Results and Discussion

### 2.1 Syntheses of Mononuclear (**2**, **3**, **4’**) and Dinuclear Cu^I^ Complexes (**4**, **5**, **6**)

**SCHEME 1:**
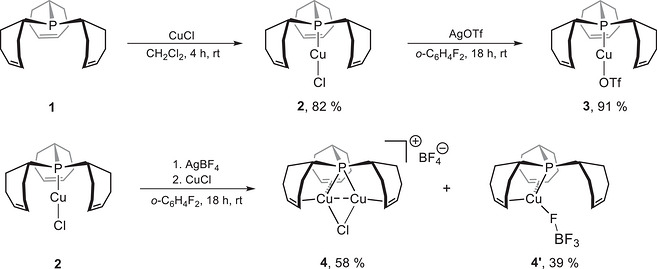
Synthesis of complexes **2**,**3**, **4** and **4’** by reaction phosphane **1** with various Cu^I^ precursor complexes.

Tris(trop)phosphine, Ptrop_3_ (**1**) [tris(dibenzo[a,d]cyclohepten‐5‐yl)phosphane; ^31^P NMR δ = −22.7 ppm; ^1^H NMR (C*H* = C*H*_trop_) δ = 6.30 ppm; ^13^C (*C*H = *C*H_trop_) δ = 125.0 ppm] was conveniently prepared according to the above cited new method [25] and reacted with Cu^I^Cl in dichloromethane for 18 h at room temperature. This gave the copper complex [Cu^I^Cl(Ptrop_3_)] (**2**), which was isolated as a yellow crystalline solid with a yield of 73 % (Scheme 1, top). A broad singlet ^31^P NMR resonance is observed for compound **2** at −31 ppm (Δν^1/2^ = 120 Hz) at room temperature, which narrows down to Δν^1/2^ = 91 Hz at ‐40°C. This phenomenon is commonly observed for copper phosphane complexes and attributed to the coupling of the ^31^P nucleus to the two NMR active quadrupolar isotopes, ^63^Cu and ^65^Cu. A ^1^H/^31^P HMBC spectrum of complex **2** (see Figure S13) shows no cross peaks between the olefinic protons (δ = 6.60 ppm) and the resonance of the ^31^P nucleus, which agrees well with the observations made for the analogous Ag^I^ and Au^I^ complexes indicating only weak interactions between the metal center and the C═C_trop_ unit [24].

In the next step, **2** was reacted with AgOTf in *o*‐C_6_H_4_F_2_ or CH_2_Cl_2_ at room temperature whereby a clean anion exchange reaction occurred to give the neutral copper complex [Cu^I^(OTf)(Ptrop_3_)] (**3**) in 91% yield (Scheme 1, top). In solution, the ^1^H NMR resonances of the C═C_trop_ unit are observed as singlet at 6.72 ppm and in the ^13^C{^1^H} as doublet at 129.4 ppm (^4^ *J*_P,C_ = 5.9 Hz). The slight shift to higher frequencies compared to the uncoordinated ligand (*vide supra*) can be taken as indication for a weak interaction with the Cu^I^ center. With the idea to strengthen the Cu^I^ C═C_trop_ interaction, **2** was reacted with silver salts with rather weakly coordinating anions such as Ag(BF_4_) or Ag(PF_6_). However, no defined complexes could be isolated but by chance the binuclear complex **4** was crystallized and investigated by X‐ray diffraction (XRD) methods (*vide infra*). In an attempt to obtain a better yield, **2** was reacted with AgBF_4_ in *o*‐C_6_H_4_F_2_ and a second equivalent of Cu^I^Cl was added subsequently, which indeed gave the bimetallic copper complex [Cu_2_(μ_2_‐Cl)(μ_2_‐Ptrop_3_)]BF_4_ (**4**) containing a μ_2_‐phosphane and a μ_2_‐chlorido ligand. Complex **4** is obtained together with the mononuclear neutral copper complex [Cu(κ^1^‐F‐BF_4_)(Ptrop_3_)] (**4’**) (bottom Scheme 1) from which it could be separated by fractional crystallization in 58% yield as yellow crystalline powder (complex **4’** can be obtained from the supernatant solution as yellow solid in about 39% yield).

Re‐dissolving the crystallised binuclear complex **4** in [D_2_]DCM leads to a turbid mixture containing a white precipitate, which proves to be Cu^I^Cl. In solution, the ^1^H NMR resonances of complex **4** and **4’** are detected indicating partial decomposition of [Cu_2_(μ_2_‐Cl)(μ_2_‐Ptrop_3_)]BF_4_ (**4**) → [Cu(κ^1^‐F‐BF_4_)(Ptrop_3_)] + Cu^I^Cl. Crystallization from this solution yielded analytical pure complex **4’** and **4** again with a similar yield as described before. Compound **4** and **4’** display rather broad ^31^P{^1^H} NMR resonances, δ (**4**) = 43.9 ppm (Δν^1/2^ = 65 Hz) and δ (**4’**) = 55.5 ppm (Δν^1/2^ = 160 Hz), indicating apart from ^63/65^Cu quadropolar coupling a fluctional behaviour of the trop moiety (for variable temperature NMR spectra see the Supporting Information). In the ^1^H NMR spectrum, the six olefinic protons of **4** are observed at δ = 6.72 ppm and for **4’** at δ = 6.83 ppm, which is indeed slightly high‐frequency shifted with respect to **2** and **3**.

**SCHEME 2:**
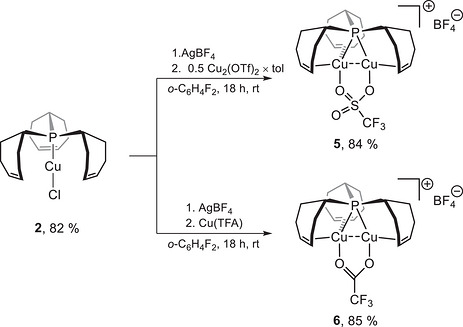
Synthesis of complex **5** and **6**.

In order to obtain well defined and more stable dinuclear complexes, we turned to copper salts with potentially more stabilizing bidentate anions as co‐ligands such as triflate, CF_3_SO_3_^−^ (OTf^−^), or trifluoroacetate, CF_3_CO_2_^−^ (TFA^−^). Indeed, when complex **2** was first reacted with AgBF_4_ followed by addition of [Cu_2_(OTf)_2_ × tol] or [Cu(TFA)] in *o*‐C_6_H_4_F_2_ as solvent, the clean formation of the desired binuclear μ_2_‐P bridged complexes [Cu_2_(μ_2_‐Ptrop_3_)(μ_2_‐κ^2^ ‐O,O’‐OTf)]BF_4_ (**5**) and [Cu_2_(μ_2_‐Ptrop_3_)( μ_2_‐κ^2^ ‐O,O’‐TFA)]BF_4_ (**6**) is observed (Scheme 2). Note that in none of the reactions shown in Schemes 1 or 2, the formation of a heteronuclear complex with a Cu‐Ag interaction was observed and also the synthesis of a related dinuclear complex with Ag‐Ag interaction failed.

**FIGURE 2:**
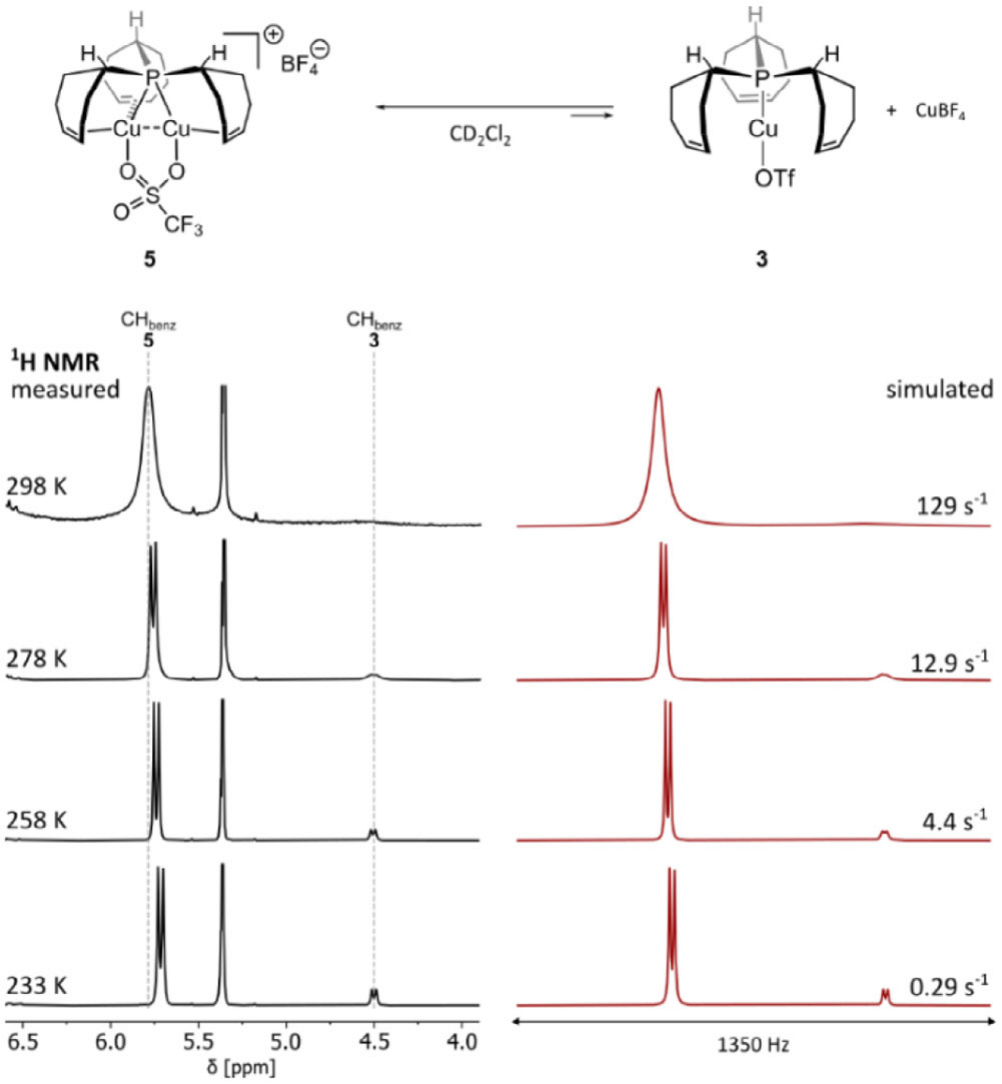
Equilibrium between dinuclear complex **5** and mononuclear complex **3**. Selected parts of ^1^H NMR spectra of **5** at different temperatures.

In **5** and **6**, the ^1^H and ^13^C NMR data again indicate a weak interaction between the CH═CH_trop_ unit and the Cu^I^_2_ core: Both, the ^1^H and ^13^C resonances show small coordination shifts, Δ*δ* = δ_complex_ – δ_ligand,_ which are slightly positive in the ^1^H NMR spectra [**5**: Δδ(^1^H) = 0.41 ppm; **6**: Δδ(^1^H) = 0.28 ppm] and slightly negative in the ^13^C NMR spectra [**5**: Δδ(^13^C) = −5.3 ppm; **6**: Δδ(^13^C) = −5.0 ppm]. Moreover, these resonances appear as singlets indicating a rapid fluxional behavior whereby the coordinated dibenzo[a,d]cyclohepten‐5‐yl (trop) units mutually change their coordination modes as bis‐η_2_‐C═C_trop_ at Cu1 and mono‐η_2_‐C═C_trop_ at Cu2 (a type of “flip‐flop” movement). Another interesting phenomenon is seen in the resonances of the benzylic proton at the bow of the central seven membered ring. In the dinuclear μ_2_‐P bridging copper complexes [Cu_2_(trop_3_P)(OTf)]BF_4_ (**5**) and [Cu_2_(trop_3_P)(TFA)]BF_4_ (**6**) this ^1^H_benz_ resonance is significantly shifted to higher frequencies to about δ(^1^H) = 5.61 ppm when compared to uncoordinated **1** [δ(^1^H) = 4.16 ppm]. Moreover, in **5** a broad singlet is observed while a sharp doublet is seen for **6** (^2^ *J*_P,H_ = 14.9 Hz). Variable temperature NMR measurements of CD_2_Cl_2_ solutions of **5** show that at low temperature (−40°C) the broad signal is resolved into a sharp doublet at 5.67 ppm (^2^ *J*_P,H_ = 14.3 Hz) (see left side of Figure 2 and Supporting Information). In addition, signals of low intensity are detected, which are assigned to those of complex **3**. These observations can be interpreted by assuming that there is an equilibrium between the bimetallic complex **5**, mono‐nuclear complex **3**, and (solvated) Cu(BF_4_). This equilibrium lies clearly to the side of the binuclear complex and a simulation of the spectra using a line shape analysis (see right side of Figure 2) allows to roughly estimate the activation parameters for this process: An Arrhenius‐plot allows to approximate the activation energy to be *E_a_* = 12.4 kcal/mol, while an Eyring plot delivered the activation parameters $\Delta H^{\neq }$ = 11.9 kcal/mol, $\Delta S^{\neq }$ = −9.61 cal/(molK), and $\Delta G^{\neq }$ = 14.7 kcal/mol at 298 K. The thermodynamic parameters of this equilibrium cannot be determined (because the aggregation state of Cu(BF_4_) in the solution is unknown).

### 2.2 Structures of Mononuclear (**2**, **3**, **4’**) and Dinuclear Cu^I^ Complexes (**4**, **5**, **6**)

**FIGURE 3:**
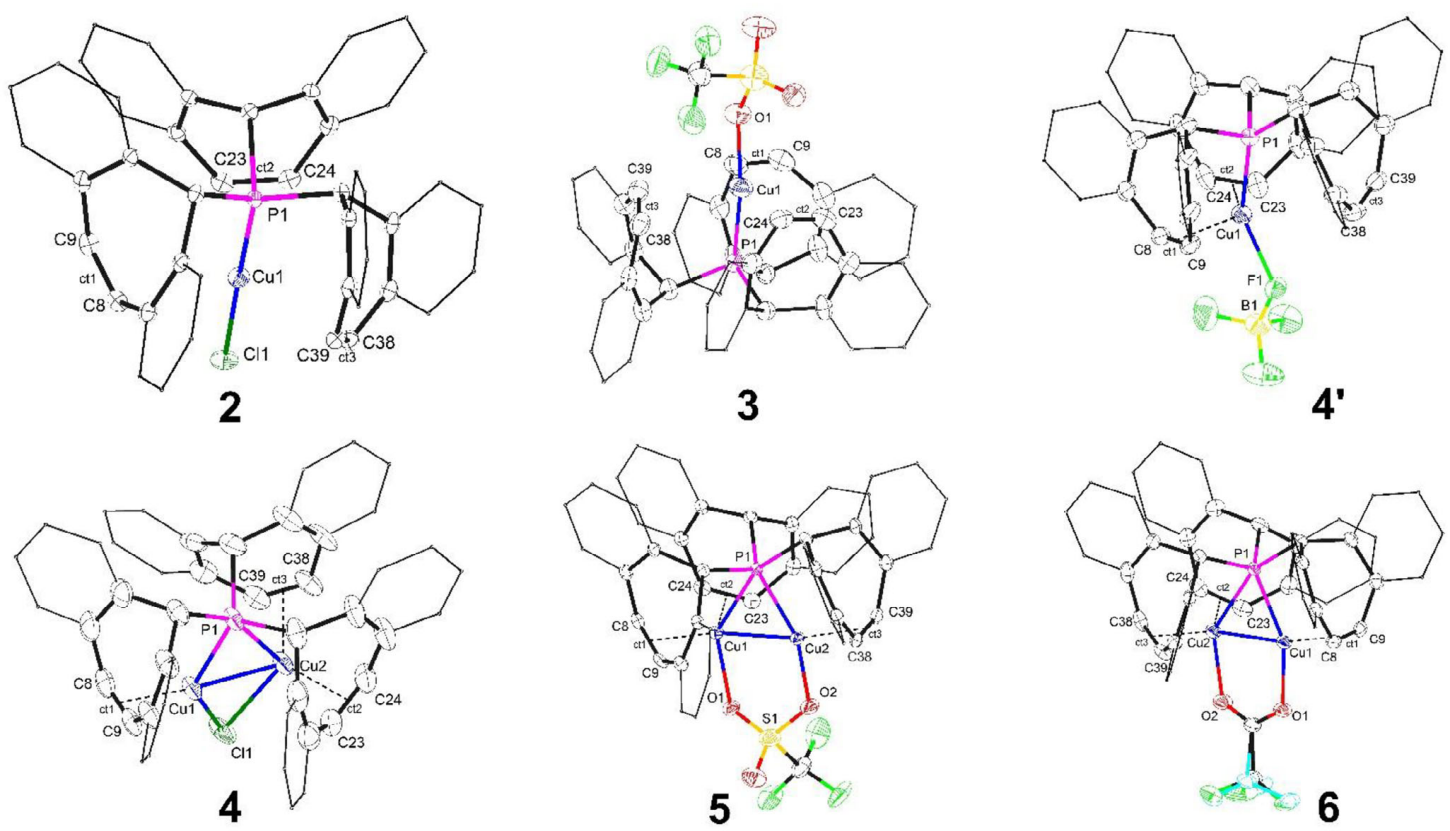
Molecular structure of complexes **2** – **6** with displacement ellipsoids drawn at 50% probability level. If a structure contains more than one crystallographically independent molecule (**3**, **4’**), only one of them is shown. H atoms, co‐crystallised solvent molecules and counter ions are omitted for clarity. The minor component of a disordered CF_3_‐group in **6** is shown in turquoise. ct1, ct2 and ct3 are the centroids of the coordinated C8═C9, C23═C24 and C38═C39 bonds, respectively. Selected bond distances [Å] and angles [deg] are given in Table 1.

The structures of **2**, **3**, **4’**, **4**, **5** and **6** were determined by X‐ray diffraction (XRD) methods [26]. Plots of the structures are shown in Figure 3 and pertinent bond lengths [Å] and angles [°] are given in Table 1. In complex **2**, the phosphorus center of the Ptrop_3_ ligand, the Cu^I^ center, and the chlorido ligand form an almost linear arrangement P1‐Cu1‐Cl1: 176.32(3)°. The Cu1‐Cl1 [2.1604(8) Å] and Cu1‐P1 [2.1756(8) Å] distances are almost identical and within the range of previously reported values for closely related complexes [22, 23].

**TABLE 1:** Selected bond distances [Å] and angles [°] for complexes **2** – **6**, ct = centre of coordinated multiple bond.

complexes	2	3^b^	4’^b^	4	5	6
Cu1‐P1	2.1756(8)	2.167(2)	2.1912(11)	2.2592(19)	2.2008(5)	2.2267(5)
Cu2‐P1				2.645(2)	2.5635(5)	2.5323(5)
Cu···Cu				2.4401(13)	2.4573(4)	2.4320(4)
Cu1‐X^a^ Cu2‐X	2.1604(8)	1.967(6)	2.110(3)	2.2225(19) 2.3392(18)	1.9540(16) 2.1685(15)	1.9552(14) 2.0747(15)
Cu1‐C8	2.866	2.910	2.261	2.181	2.234(2)	2.2542(19)
Cu1‐C9	3.233	2.626	2.345	2.180	2.265(2)	2.2744(19)
Cu1/2‐C23	2.672	2.989	2.291	2.170	2.1806(19)	2.159(2)
Cu1/2‐C24	2.870	2.676	2.404	2.183	2.1577(19)	2.227(2)
Cu1/2‐C38	3.074	3.055	3.434	2.189	2.2485(19)	2.182(2)
Cu1/2‐C39	3.799	2.709	3.578	2.133	2.1358(18)	2.188(2)
C8═C9	1.341(4)	1.342(13)	1.352(6)	1.367(11)	1.367(3)	1.362(3)
C23═C24	1.351(4)	1.367(12)	1.347(6)	1.358(11)	1.373(3)	1.367(3)
C38═C39	1.344(4)	1.334(12)	1.338(6)	1.371(10)	1.371(3)	1.370(3)
P1‐Cu1‐X^a^	176.32(3)	167.71(19)	140.33(8)			
P1‐Cu1‐Cl1/O1				128.34(8)	159.64(5)	154.28(5)
P1‐Cu2‐Cl1/O2				107.92(6)	136.93(4)	138.49(4)

^a^ **2**: X = Cl; **3**: X = OTf; **4’**: X = BF_4_.

^b^ averaged value.

The distances between the centroids of the C═C_trop_ unit and Cu1 are long and vary over a large range from 2.689 [Cu1‐ct2(C23═C24)] to 3.390 Å [Cu1‐ct3(C38═C39)] and together with marginally elongated C═C_trop_ bonds (av. 1.345 Å vs. 1.33 Å in **1** ^22^) indicate only a very weak interaction with the Cu^I^ center in accord with the conclusions drawn from the NMR spectra.

Crystals of **3** contain two independent molecules in the unit cell; the average Cu1‐O1 bond length is 1.97 Å long and within the expected range of related copper complexes with terminal coordinated sulfate or sulfonate groups (1.9 – 2.4 Å) [27]. The P‐Cu‐O angle (average 168°) deviates slightly from linearity. Compared to **2**, the distances between Cu1 and the centroids of the C═C_trop_ are indeed somewhat shorter (2.504 – 2.875 Å) but they remain long and in combination with marginally elongated C═C_trop_ bond lengths indicate no significant interaction with the copper center.

The coordination sphere around the Cu(1) center in **4’** is best described as a distorted disphenoid. The Cu1‐P1 distance [2.1835(9) Å] is in the same range as in **2** and **3**. However, in **4’** two of the Cu‐ct distances are shorter (Cu‐ct1 = 2.222 Å, Cu‐ct2 = 2.317 Å) while the third is again very long (3.422 Å). Likewise, the C8═C9 [1.360(6) Å] and C23═C24 [1.351(5) Å] distances are marginally longer than the C38═C39 bond [1.349(6) Å] indicating that an interaction between Cu and C═C units may become noticeable at Cu‐ct distances below 2.5 Å. The fourth contact to the Cu^I^ center is made by the BF_4_^−^ anion, which binds with one fluorine center in a terminal fashion to Cu1 [Cu‐FBF_3_): 2.107(2) Å].

The solid‐state structures of the binuclear copper complexes **4**, **5**, and **6** are shown in bottom row of Figure 3. In all complexes the phosphorus center adopts an unsymmetrical μ_2_‐bridging mode with one short Cu‐P bond, which forms between the phosphorus center and the copper center Cu1 of lower coordination number. In all complexes, the Cu1 resides in a distorted trigonal planar coordination sphere (not taking the Cu···Cu interaction into account) while Cu2 is located in a distorted tetrahedral coordination environment. The Cu1‐P distances range from 2.2008(5) Å in **5** to 2.258(2) Å in **4** (average: Cu1‐P = 2.23 Å), while the Cu2‐P distances are significantly longer, ranging from 2.5323(5) Å in **6** to 2.642(12) Å in **4** (average: Cu2‐P = 2.58 Å; Δ(Cu‐P)_av_ = 0.35 Å). If one defines a bond difference Δ(M‐P) of < 0.1 Å as symmetric and of > 0.1 Å as asymmetric, then to date there are almost as many Cu^I^ complexes with symmetric as there are ones with asymmetric μ_2_‐ and μ_3_‐bridging phosphanes [5, 6, 7, 8, 10, 11, 15] indicating that the total energies of the electronic ground states are likely small [7]. The Cu‐Cu distances vary little and are in the range of 2.4320(4) – 2.4573(4) Å, which is on the shorter side of typical d^10^‐d^10^ Cu^I^‐Cu^I^ (cuprophilic) interactions [12, 13, 14, 28] and well within the sum of the van der Waals radii of Cu (2.80 Å) [29]. Like the Cu1‐P bonds, all Cu1‐X heteroatom bonds (X = Cl in **4**, L = O in **5**, **6**) are shorter when compared to Cu2‐X, which are about 10% longer (for data see Table 1). Especially noteworthy are the Cu‐ct distances in the binuclear complexes **4** – **6**, which are significantly shorter and in a narrower range (2.007 – 2.160 Å) compared to the mononuclear complex **D** (Cu‐ct 2.335, 2.299 Å). But all Cu‐C distances in **4** – **6** (see Table S13) are still significantly longer than Cu‐C distances in other copper alkene complexes [27, 30, 31]. Likewise the slightly more elongated C═C_trop_ bonds (av. 1.371 Å) indicate a more pronounced interaction with the Cu^I^ centers in the binuclear complexes.

### 2.3 DFT Calculations on Mononuclear Complex [Cu(Ptrop_3_)OTf] (**3**) and Dinuclear Complex [Cu_2_(μ_2_‐Ptrop_3_)(OTf)]BF_4_ (**5**)

The electronic structures of the mononuclear complex [Cu(Ptrop_3_)OTf] (**3**) and related dinuclear complex [Cu_2_(μ_2_‐Ptrop_3_)(OTf)]BF_4_ (**5**), which reversibly interconvert via the uptake or release of a Cu^+^ ion, were investigated by DFT methods [32]. The calculated bond data are listed in Table 2 and the agreement between the experimental and calculated data is very good.

**TABLE 2:** The selected bond distance (Å), Wiberg bond index (WBI), NBO donor‐acceptor interaction energies *E* ^(2)^ (kcal mol^−1^) of complex **3** and **5**.

Complexes	3			5		
		WBI	*E* ^(2)^ ^a^		WBI	*E* ^(2)^ ^a^
Cu1‐P1	2.19	0.33	52.1	2.24	0.34	48.3
Cu2‐P1				2.58	0.16	18.0
Cu···Cu				2.46	0.12	4.7(1.9)
Cu1‐C8 Cu1‐C9	2.65 2.93	0.03 0.02	3.9(0.8)	2.36 2.29	0.07 0.07	10.8(6.3)
Cu1/2‐C23 Cu1/2‐C24	2.92 2.60	0.02 0.03	3.3(1.0)	2.39 2.17	0.08 0.11	15.1(4.9)
Cu1/2‐C38 Cu1/2‐C39	3.02 2.64	0.02 0.03	3.0(0.9)	2.25 2.18	0.12 0.10	16.7(7.1)
Cu1‐O1	1.96	0.15	24.7	1.95	0.15	30.4
Cu2‐O2				2.10	0.10	14.7

^a^ σ/π‐Type donation to the metal and π‐back donation from the metal (in brackets).

The reaction [Cu^I^(Ptrop_3_)(OTf)] (**3**) + Cu^I^BF_4_ → [Cu^I^_2_(μ_2_‐Ptrop_3_)(μ_2_‐κO‐κO’‐OTf)]BF_4_ (**5**) was calculated at the B3LYP/Def2‐TZVP‐SMD(dichloromethane)//B3LYP‐D3(BJ)/Def2‐SVP level of theory, including SMD solvent model for DCM. In accordance with the experiment, this reaction is exergonic and the calculated free Gibbs energy ∆G^calc^ is −33.1 kcal·mol^‒1^. The rather high exothermicity is likely a result of the high energy of Cu^I^BF_4_, which in the calculation is assumed to be a solvated mononuclear [Cu(κ^3^ ‐F,F′,F′′‐BF_4_)] complex (see the Supporting Information for details).

The thermochemistry of the reaction [Cu^I^(Ptrop_3_)(OTf)] (**3**) + [Cu^I^_2_(μ_2_‐PMe_3_)( μ_2_‐κO‐κO’‐OTf)]^+^ → [Cu^I^_2_(μ_2_‐Ptrop_3_)(μ_2_‐κO‐κO’‐OTf)]^+^ (**5^+^**) + [Cu(PMe_3_)(OTf)] was calculated, which involves the hypothetical dinuclear complex cation [Cu^I^_2_(μ_2_‐PMe_3_)( μ_2_‐κO‐κO’‐OTf)]^+^ with a very asymmetrically bound μ_2_‐PMe_3_ bridge (Cu1‐P 2.21 Å; Cu2‐P 3.06 Å) as carrier of a Cu^I^ cation (for details see the Supporting Information). The free Gibbs energy of this reaction is – as expected –less substantial with ∆G^calc^ of −19.5 kcal·mol^‒1^. But yet its significant exergonicity underscores the stabilizing effect of the trop substituents with its olefinic binding groups for the formation of dinuclear complex **5**.

We estimated the deformation energy, which is necessary to convert Ptrop_3_ from its ground state structure to the one it adopts as ligand either in the mononuclear complex **3** or dinuclear complex **5**. For this, the geometry of Ptrop_3_ was optimized to give the energy of the electronic ground state *E*°(Ptrop_3_). The total energies of the “deformed” Ptrop_3_ moieties with structures identical to those in either **3** [*E* ^3^(Ptrop_3_)] or **5** [E^5^(Ptrop_3_)] were determined by single‐point calculations. The difference *E*°(Ptrop_3_) − *E* ^X^(Ptrop_3_) (X = **3**, **5**) was taken as estimate for the energy required to widen Ptrop_3_ to be able to host a Cu^I^ ion or a Cu^I^_2_ unit. Accordingly, the structural change of P(trop)_3_ in **3** is accompanied by a negligible energy of +2.8 kcal mol^−1^ while a deformation energy of +11.0 kcal mol^−1^ is required to achieve the structure in **5** [Σ°(P) in Ptrop_3_ = 295; in **3** = 309.9; in **5** = 310.2).

In order to characterize the bond between the Cu^I^ centers and the Ptrop_3_ ligand in more detail, we calculated the Wiberg bond indices (WBIs) and also the donor‐acceptor interaction energies based on a Natural Bond Orbital (NBO) analysis [33]. These results are listed in Table 2.

**FIGURE 4:**
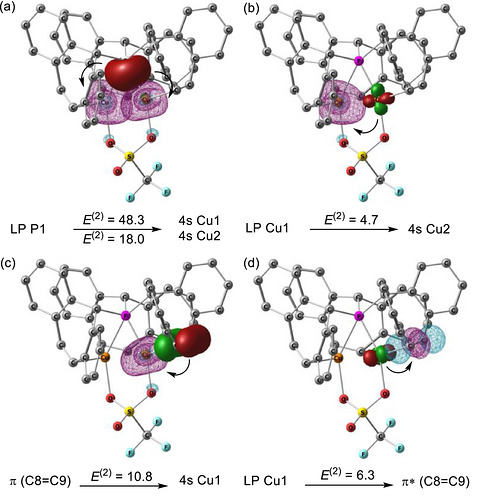
Second order perturbation analysis of **5** (isovalue = 0.08).

The donor‐acceptor energy obtained from a second order perturbation analysis *E* ^(2)^ for the donation of the lone pair of P1 to the valence 4s‐orbital of Cu1 is by far the strongest and amounts to 52.1 kcal mol^−1^ in complex **3**, which is in agreement with the shortest Cu‐P1 distance (2.19 Å) and the largest WBI (0.33) of all complexes. As expected, the two Cu‐P interactions in the binuclear complex **5** are very different. While the short Cu1‐P1 (2.24 Å) has a WBI comparable to the Cu1‐P1 bond in **3** and also *E* ^(2)^ is very similar (48.3 kcal mol^−1^), the second interaction between Cu2 and P1 (with 2.58 Å) has a WBI of only 0.16 and its interaction energy E^(2)^ = 18.0 kcal mol^−1^ is only about a third (Figure 4a).

The Cu···Cu interaction in **5** shows a WBI of 0.12 (in the range of the Cu olefin interactions in this complex) while the *E* ^(2)^ value (6.6 kcal mol^−1^) is small but not negligible (Figure 4b). The interactions between Cu^I^ and the olefins are much weaker than the Cu‐P interactions and the WBIs do not exceed 0.03 in **3** or 0.12 in **5**. Note, however, that the interaction energies *E* ^(2)^ for the total Cu^I^ olefin interaction in **5** (Figure 4c) is considerably higher than that in **3**. Especially the π‐type back donation is more significant in **5** (Figure 4d). The μ_2_‐κO,κO’‐OTf ligand also contributes significantly to the stability of the dinuclear complex **5** and in sum (45.2 kcal mol^−1^) correspond to the Cu1‐P interaction [about 25% of the total *E* ^(2)^]. Note that the total *E* ^(2)^ in **5** (178.9 kcal mol^−1^) is 89.3 kcal mol^−1^ higher than that in **3** (89.6 kcal mol^−1^), which is more than enough to compensate the deformation energy of the ligand (11.0 kcal mol^−1^) in **5**. Overall, the results from the DFT calculation fully support the exergonic formation of **5** from **3** and a Cu^I+^ source.

## 3 Conclusion

In contrast to previous reports, not only phosphanes with additional dangling relatively strong σ‐donor groups (pyridines) can be employed as polydentate ligands for the stabilization of dinulear Cu^I^ complexes with μ_2_‐bridging phosphanes, but ligands with relatively weak σ‐donor but rather strong π‐acceptor properties such as olefins are also suitable for this purpose. Preorganisation effects are likely important for efficient Cu^I^ olefin interactions [30, 34] and this seems also to be true for the formation of the binuclear Cu^I^ complexes reported in this paper. The rigid central seven‐membered concavely shaped binding sites of the trop units do not interact strongly with the Cu^I^ center in the mononuclear complex (only about 10% of the total interaction energy *E* ^(2)^ are due to the Cu^I^ olefin interactions) while in the binuclear complex they make up to a quarter of *E* ^(2)^, which on one side is due to the closer proximity of the metal centers to the C = C binding sites and to the other to the increased electron density of the Cu^I^_2_ core, which specifically enhances the M‐C═C π‐back donation. However, all interactions apart from Cu‐P are relatively weak so variations of bond lengths and angles reflect shallow energy surfaces on which the complexes reported here can easily adopt slightly different structures. For the future, it shall be interesting to convert complexes like **4** – **6** into functional entities by, for example, replacing the OTf or TFA ligand into one that can be chemically modified. One such possibility may the use of nitrites or nitrates, which eventually could be catalytically deoxygenized and converted to amine derivatives. At present only phosphanes with additional dangling binding sites (nitrogen, olefins) give dinuclear Cu^I^ complexes with μ_2_‐bridging phosphanes stable enough to be isolated. This is in contrast to complexes with a Cu^I^_2_ core and phosphinines as symmetrically bridging ligands, which are stable as such [16, 17]. Although the calculated structure of [Cu^I^_2_(μ_2_‐PMe_3_)( μ_2_‐κO‐κO’‐OTf)]^+^ lacks experimental evidence, the challenge of synthesizing and investigating these type of complexes with very unsymmetrically bridging alkyl phosphines shall be taken up.

## Conflicts of Interest

The authors have nothing to report.

## Supporting information




**Supporting File**: chem71085‐sup‐0001‐SuppMat.docx
