# Supplementary material for: Dinuclear Copper(I) Complexes Featuring Metal–Metal Interactions and a μ2‐Bridging Phosphane With Additional Olefinic Binding Sites
Source: Chemistry. 2026 May 3;32(27):e71085. doi: 10.1002/chem.71085 (PMC13380376; doi:10.1002/chem.71085)
Supplement: Supplementary file 1 — Supporting File: chem71085‐sup‐0001‐SuppMat.docx [file CHEM-32-e71085-s001.docx]

**Synthesis of Unusual Bimetallic Olefinic Copper Complexes featuring a μ_2_-P Bridging Coordination Mode**

Daniel Himmelbauer,^a^ Jan Oswald,^a^ Michael Wörle,^a^ René Verel,^a^ Zoltán Benkő,^b^ and Hansjörg Grützmacher*^*a^*

***Corresponding authors

*a. Department of Chemistry and Applied Biosciences, ETH Zurich, Vladimir-Prelog-Weg 1, CH-8049 Zurich
E-mail:* [*hgruetzmacher@ethz.ch*](mailto:hgruetzmacher@ethz.ch)

*b. Department of Inorganic and Analytical Chemistry, Budapest University of Technology and Economics, 1111 Budapest, Műegyetem rakpart 3, Hungary
E-mail:* [*benko.zoltan@vbk.bme.hu*](mailto:benko.zoltan@vbk.bme.hu)

Supporting Information

[1	Experimental Procedures	3](#_Toc158811945)

[1.1	General Considerations	3](#_Toc158811946)

[1.2	Synthetic Procedure	3](#_Toc158811947)

[2	NMR Spectra	7](#_Toc158811948)

[2.1	VT-NMR Experiments	41](#_Toc158811949)

[3	MS Spectra	44](#_Toc158811950)

[4	IR-ATR Spectra	50](#_Toc158811951)

[5	UV-VIS Spectra	51](#_Toc158811952)

[6	Crystallographic Data	52](#_Toc158811953)

[7	References	65](#_Toc158811954)

# 1 Experimental Procedures

## 1.1 General Considerations

All air- and moisture-sensitive manipulations were carried out using standard vacuum line Schlenk techniques or in an MBraun inert atmosphere dry-box containing an atmosphere of purified argon. Deuterated solvents (CD_2_Cl_2_, THF-d8, etc.) were purchased from Cambridge Isotope Laboratories and dried over molecular sieves (4 Å). All glassware was either stored in a 130 °C oven or dried under vacuum using a heat gun and was degassed prior to. Syringes and needles were purchased from Braun. CHROMAFIL Xtra PTFE-45/25, PALL Acrodisc GHP 13 mm membrane 0.45 and 0.20 µm syringe filters were used. Solvents were used directly from a solvent purification system and stored over molecular sieves. 1,2-difluorobenzene were stored over P_4_O_10_ for several days and then distilled. TropCl^[1]^ and H_3_P$\to$AlCl_3_^[2]^ were prepared according to the literature.

^1^H, ^13^C, ^11^B, ^19^F, ^31^P and ^63^Cu NMR spectra were recorded on Bruker AVANCE-200, AVANCE-300, AVANCE-400, and AVANCE-500 spectrometers. Multiplicities are abbreviated as singlet (s), doublet (d), triplet (t), quartet (q), multiplet (m) and broad (br). NMR spectra were referenced to TMS (^1^H, ^13^C), CFCl_3_ (^19^F), 85 % H_3_PO_4_ aqueous solution (^31^P), and 15 % solution of BF_3_^.^OEt_2_ in CDCl_3_ (^11^B).

ATR and solution infrared spectra were collected on a Bruker-alpha FT-IR-spectrometer. FT-IR spectra were collected in the solid state with the ATR technique on a Bruker Alpha inside an MBraun glovebox.

X-ray diffraction experiments were performed on a Rigaku XtaLAB Synergy-S diffractometer, equipped with a HyPix-6000HE hybrid photon counting detector, using a microfocus tube (Cu, λ = 1.54184 Å). Suitable crystals were covered in a glove box with polybutene oil, selected using a microscope using polarized light, transferred on a MiTeGen MicroMount and mounted under a cold nitrogen stream. Data-sets were collected at 100 K. The data collection and reduction were performed using the Rigaku CrysAlisPro Software. Structure solution and refinement were performed with SHELXT^[3]^ and SHELXL^[4]^, respectively, embedded in Olex2^[5]^. Except noted otherwise, all atoms heavier than hydrogen were refined with anisotropic displacement parameters, while hydrogen atoms were placed at calculated positions and were refined according to the riding model.

UV-VIS-spectra were recorded on an Agilent Cary5000. Mass Spectra were recorded on a Thermo Finnigan TS Quantum. Elemental analyses were performed at the Mikrolabor of ETH Zürich.

## 1.2 Synthetic Procedures

*Trop_3_P* (**1**)^[6]^. TropCl (11.2 g, 50.0 mmol) was dissolved in dry mesitylene (100 ml) followed by the slow addition of H_3_P$\to$AlCl_3_ (16.5 ml, 16.5 mmol) at 0 °C. The mixture was left stirring at room temperature for 24 h Then the solvent was evaporated under reduced pressure. The product was liberated by suspending it in concentrated sodium bicarbonate solution and isolated by extracting it twice with methylene chloride. The combined organic phases were dried with sodium sulfate and concentrated to dryness. The crude product was purified by washing it with small amounts of toluene. Then it was washed three times with *n*-pentane and dried under high vacuum yielding 7.3 g (73 %) of the product in pure form as a white solid. If necessary, **1** can be further purified by recrystallisation from toluene. For spectroscopic data see ref.^[6]^.

*Synthesis of [Cu(trop_3_P)Cl]* (**2**). **1** (1.8 g, 3.0 mmol) and CuCl (0.3 g, 3.0 mmol) were put into a 100 ml Schlenk flask and CH_2_Cl_2_ (25 ml) was added. The reaction mixture was stirred 18 h at room temperature becoming yellow and slightly turbid. The turbid solution was filtered over a syringe filter and layered with *n*-hexane (40 ml). After 7 d the crystalline product was collected, washed with *n*-pentane (3 × 7 ml) and dried under high vacuum at 55 °C overnight yielding 1.6 g (76 %). Elemental Analysis for C_45_H_33_ClCuP (703.73 g/mol) found (calc.) [%]: C 76.58 (76.80), H 4.72 (4.73). MS (ESI^+^, MeOH): m/z calcd for C_45_H_33_CuP [M−Cl]^+^ 667.16, found 667.19. ^1^H NMR (500 MHz, CD_2_Cl_2_, 25 °C): δ = 4.40 (d, ^2^*J*_P,H_ = 12.2 Hz, 3 H, H_benz_), 6.55-6.57 (m, 6 H, H_aryl_), 6.60 (s, 6 H, H_olef_), 6.91-6.92 (m, 6 H, H_aryl_), 7.06-7.12 (m, 12 H, H_aryl_) ppm. ^1^H NMR (500 MHz, CD_2_Cl_2_, -40 °C): δ = 4.37 (d, ^2^*J*_P,H_ = 12.3 Hz, 3 H, H_benz_), 6.53-6.54 (m, 6 H, H_aryl_), 6.56 (s, 6 H, H_olef_), 6.86-6.88 (m, 6 H, H_aryl_), 7.03-7.08 (m, 12 H, H_aryl_) ppm. ^13^C{^1^H} NMR (126 MHz, CD_2_Cl_2_, 25 °C): δ = 54.6 (d, ^1^*J*_P,C_ = 9.5 Hz, CH_aryl_), 126.8 (s, CH_benz_), 129.3 (d, *J*_P,C_ = 4.8 Hz, CH_benz_), 129.6 (s, CH_benz_), 132.2 (d, ^4^*J*_P,C_ = 4.5 Hz, CH_olef_), 135.2 (d, *J*_P,C_ = 4.5 Hz, C_benz_), 136.2 (d, *J*_P,C_ = 2.2 Hz, C_benz_) ppm. ^31^P{^1^H} NMR (203 MHz, CD_2_Cl_2_, 25 °C): δ = −31.0 (s, trop_3_P) ppm. ^31^P{^1^H} NMR (203 MHz, CD_2_Cl_2_, -40 °C): δ = −33.4 (s, trop_3_P) ppm.

Synthesis of [Cu(trop_3_P)(OTf)] (**3**): To a solution of [Cu(trop_3_P)Cl] (20 mg, 0.028 mmol) in *o*-C_6_H_4_F_2_ (1 ml) was added AgOTf (8 mg, 0.03 mmol). The reaction mixture was stirred overnight at room temperature. The next day the solution was filtered, and all volatiles were removed under high vacuum, forming a yellow microcrystalline yellow solid. The product was dried 3 h under high vacuum at room temperature yielding 21 mg (91 %) of **3** as pale yellow solid. Elemental Analysis for C_46_H_33_CuF_3_O_3_PS (817.34 g/mol) found (calc.) [%]: C 67.40 (67.60), H 4.20 (4.07). MS (ESI^+^, MeOH): m/z calcd for C_45_H_33_CuP [M−OTf]^+^ 667.16, found 667.19.^1^H NMR (500 MHz, CD_2_Cl_2_, 25 °C): δ = 4.46 (d, ^2^*J*_P,H_ = 12.4 Hz, 3 H, H_benz_), 6.63-6.65 (m, 6 H, H_aryl_), 6.72 (s, 6 H, H_olef_), 6.87-6.89 (m, 6 H, H_aryl_), 7.04-7.06 (m, 12 H, H_aryl_) ppm. ^1^H NMR (500 MHz, CD_2_Cl_2_, -40 °C): δ = 4.44 (d, ^2^*J*_P,H_ = 12.5 Hz, 3 H, H_benz_), 6.61-6.63 (m, 6 H, H_aryl_), 6.67 (s, 6 H, H_olef_), 6.81-6.83 (m, 6 H, H_aryl_), 6.98-7.04 (m, 12 H, H_aryl_) ppm. ^13^C{^1^H} HNMR (126 MHz, CD_2_Cl_2_, 25 °C): δ = 54.2 (d, ^1^*J*_P,C_ = 9.5 Hz, CH_aryl_), 126.9 (s, CH_benz_), 129.1 (d, *J*_P,C_ = 5.0 Hz, CH_benz_), 129.6 (s, CH_benz_), 130.1 (s, CH_benz_), 130.3 (d, ^4^*J*_P,C_ = 5.9 Hz, CH_olef_), 134.4 (d, *J*_P,C_ = 5.0 Hz, C_benz_), 135.6 (d, *J*_P,C_ = 1.6 Hz, C_benz_) ppm. The ^13^C resonance of the OTf ligand could not be detected. ^13^C{^1^H} HNMR (126 MHz, CD_2_Cl_2_, −40 °C): δ = 52.9 (d, ^1^*J*_P,C_ = 10.0 Hz, CH_aryl_), 126.3 (s, CH_benz_), 128.4 (d, *J*_P,C_ = 5.0 Hz, CH_benz_), 128.9 (s, CH_benz_), 129.4 (d, ^4^*J*_P,C_ = 5.9 Hz, CH_olef_), 129.7 (s, CH_benz_), 133.4 (d, *J*_P,C_ = 4.5 Hz, C_benz_), 134.7 (s, C_benz_) ppm. The ^13^C resonance of the OTf ligand could not be detected. ^31^P{^1^H} NMR (203 MHz, CD_2_Cl_2_, 25 °C): δ = −44.3 (br, trop_3_P) ppm. ^31^P{^1^H} NMR (203 MHz, CD_2_Cl_2_, −40 °C): δ = −47.7 (br, trop_3_P) ppm. ^19^F{^1^H} NMR (471 MHz, CD_2_Cl_2_, 25 °C): δ = −78.0 (s, CF_3_) ppm. ^19^F{^1^H} NMR (471 MHz, CD_2_Cl_2_, −40 °C): δ = −78.1 (s, CF_3_) ppm.

*Synthesis of [Cu_2_(trop_3_P)(μ^2^-Cl)]BF_4_* (**4**) and *[Cu(trop_3_P)(BF_4_)]* (**4’**): To a solution of **2** (55 mg, 0.078 mmol) in *o*-C_6_H_4_F_2_ (3 ml) was added AgBF_4_ (16 mg, 0.082 mmol). After stirring for 120 min at room temperature CuCl (8 mg, 0.080 mmol) was added. The reaction mixture was stirred at room temperature overnight. The next day all volatiles were removed under high vacuum and the residue re-dissolved in CH_2_Cl_2_ (4 ml) and filtered over a syringe filter forming a yellow solution which was layered with *n*-hexane (6 ml). After 18 h the crystalline product was collected, washed with *n*-pentane (4 × 3 ml) and dried under high vacuum at 70 °C for 2 d. Yield of **4** as a yellow crystalline solid 38 mg (56 %). The supernatant was transferred into a Schlank flask and all volatiles were removed at reduced pressure. The formed yellow solid was washed with *n*-pentane (3 × 2 ml) and then dried under high vacuum for 24 h at room temperature. Yield of **4** as a yellow crystalline solid 22 mg (38 %).

[Cu_2_(trop_3_P)(μ^2^-Cl)]BF_4_ (**4**): MS (ESI^+^, CH_2_Cl_2_): m/z calcd for C_45_H_33_Cu_2_PCl [M]^+^ 765.06, found 765.05. ^1^H NMR (500 MHz, CD_2_Cl_2_, 25 °C): 5.67 (br d, ^2^*J*_P,H_ = 10.7 Hz, 3 H, H_benz_), 6.72 (s, 6 H, H_olef_), 6.95-6.96 (m, 6 H, H_aryl_), 7.04-7.07 (m, 6 H, H_aryl_), 7.16-7.22 (m, 12 H, H_aryl_) ppm. ^1^H NMR (500 MHz, CD_2_Cl_2_, −40 °C): 5.65 (d, ^2^*J*_P,H_ = 13.0 Hz, 3 H, H_benz_), 6.71 (s, 6 H, H_olef_), 6.93-6.94 (m, 6 H, H_aryl_, overlapping with **4’**), 7.00-7.03 (m, 6 H, H_aryl_, overlapping with **4’**), 7.11-7.18 (m, 12 H, H_aryl_, overlapping with **4’**) ppm. ^13^C{^1^H} NMR (126 MHz, CD_2_Cl_2_, −40 °C): δ = 54.6 (d, ^1^*J*_P,C_ = 6.3 Hz, CH_aryl_), 114.7 (s, CH_olef_), 129.5 (s, CH_benz_), 129.9 (s, CH_benz_), 130.8 (d, *J*_P,C_ = 5.5 Hz, CH_benz_), 131.7 (s, CH_benz_), 131.7 (s, C_benz_), 133.7 (s, C_benz_) ppm. ^31^P{^1^H} NMR (203 MHz, CD_2_Cl_2_, 25 °C): δ = −40.5 (s, trop_3_P) ppm. ^31^P{^1^H} NMR (203 MHz, CD_2_Cl_2_, −40 °C): δ = −43.9 (s, trop_3_P) ppm. ^19^F{^1^H} NMR (471 MHz, CD_2_Cl_2_, 25 °C): δ = −152.3 (s, BF_4_) ppm. ^19^F{^1^H} NMR (471 MHz, CD_2_Cl_2_, −40 °C): δ = −151.3 (s, BF_4_) ppm.

[Cu(trop_3_P)( BF_4_)] (**4’**): MS (ESI^+^, CD_2_Cl_2_): m/z calcd for C_45_H_33_CuP [M−BF_4_]^+^ 667.16, found 667.18. ^1^H NMR (300 MHz, CD_2_Cl_2_, 25 °C): 4.54 (d, ^2^*J*_P,H_ = 11.8 Hz, 3 H, H_benz_), 6.69-6.71 (m, 6 H, H_aryl_), 6.83 (s, 6 H, H_olef_), 6.86-6.89 (m, 6 H, H_aryl_), 6.96-7.06 (m, 12 H, H_aryl_) ppm. ^1^H NMR (500 MHz, CD_2_Cl_2_, −40 °C): 4.53 (d, ^2^*J*_P,H_ = 11.9 Hz, 3 H, H_benz_), 6.67-6.68 (m, 6 H, H_aryl_), 6.78 (s, 6 H, H_olef_), 6.81-6.83 (m, 6 H, H_aryl_), 6.92-6.95 (m, 6 H, H_aryl_), 6.98-7.01 (m, 6 H, H_aryl_) ppm. ^13^C{^1^H} NMR (75 MHz, CD_2_Cl_2_, 28 °C): δ = 54.0 (d, ^1^*J*_P,C_ = 6.8 Hz, CH_aryl_), 127.0 (s, CH_benz_), 127.6 (s, CH_olef_), 129.2 (s, CH_benz_), 129.7 (s, CH_benz_), 130.4 (s, CH_benz_), 134.0 (s, C_benz_), 135.7 (s, C_benz_) ppm. ^31^P{^1^H} NMR (121 MHz, CD_2_Cl_2_, 25 °C): δ = −55.5 (s, trop_3_P) ppm. ^31^P{^1^H} NMR (203 MHz, CD_2_Cl_2_, -40 °C): δ = −57.3 (s, trop_3_P) ppm. ^19^F{^1^H} NMR (471 MHz, CD_2_Cl_2_, −40 °C): δ = −156.9 (s, BF_4_) ppm.

*Synthesis of [Cu_2_(Ptrop_3_)(OTf)]BF_4_* (**5**): To a solution of **2** (84 mg, 0.12 mmol) in *o*-C_6_H_4_F_2_ (4 ml) was added AgBF_4_ (26 mg, 0.13 mmol). After stirring for 120 min at room temperature [Cu_2_(OTf)_2_]·tol (36 mg, 0.070 mmol) was added. The reaction mixture was stirred at room temperature overnight. The next day all volatiles were removed under high vacuum and the residue re-dissolved in CH_2_Cl_2_ (4 ml) and filtered over a syringe filter forming a yellow solution which was layered with *n*-hexane (6 ml). After 18 h the needle like crystalline product was collected, washed with *n*-pentane (4 × 3 ml) and dried under high vacuum at 70 °C for 2 d. Yield of **5** as a yellow crystalline solid 98 mg (84 %). Elemental Analysis for C_46_H_33_BCu_2_F_7_O_3_PS (967.69 g/mol) found (calc.) [%]: C 56.54 (57.10), H 3.44 (3.44). MS (ESI^+^, CH_2_Cl_2_): m/z calcd for C_46_H_33_Cu_2_PF_3_O_3_PS [M]^+^ 879.0, found 879.0. ^1^H NMR (300 MHz, CD_2_Cl_2_, 26 °C): 5.61 (br, 3 H, H_benz_), 6.71 (s, 6 H, H_olef_), 6.97-6.99 (m, 6 H, H_aryl_), 7.10-7.25 (m, 18 H, H_aryl_) ppm. ^1^H NMR (500 MHz, CD_2_Cl_2_, −40 °C): δ = 5.67 (d, ^2^*J*_P,H_ = 14.3 Hz, 3 H, H_benz_), 6.69 (s, 6 H, H_olef_), 6.97-6.98 (m, 6 H, H_aryl_), 7.09-7.12 (m, 6 H, H_aryl_), 7.16-7.22 (m, 12 H, H_aryl_) ppm. ^31^P{^1^H} NMR (203 MHz, CD_2_Cl_2_, 25 °C): δ = −41.7 (s, trop_3_P) ppm. ^19^F{^1^H} NMR (471 MHz, CD_2_Cl_2_, 25 °C): δ = −76.3 (s, CF_3_), −148.0 (s, BF_4_) ppm. ^13^C{^1^H} NMR (126 MHz, CD_2_Cl_2_, −40 °C): δ = 55.3 (d, ^1^*J*_P,C_ = 14.5 Hz, CH_aryl_), 119.7 (s, CH_olef_), 127.7 (s, CH_benz_), 129.4 (s, CH_benz_), 130.8 (d, *J*_P,C_ = 6.4 Hz, CH_benz_), 131.0 (d, *J*_P,C_ = 5.9 Hz, C_benz_), 132.1 (s, CH_benz_), 133.2 (s, C_benz_) ppm. The ^13^C resonance of the OTf ligand could not be detected. ^31^P{^1^H} NMR (203 MHz, CD_2_Cl_2_, −40 °C): δ = −43.4 (s, trop_3_P) ppm. ^19^F{^1^H} NMR (471 MHz, CD_2_Cl_2_, −40 °C): δ = −76.5 (s, CF_3_), −147.4 (s, BF_4_) ppm.

*Synthesis of [Cu_2_(trop_3_P)(TFA)]BF_4_* (**5**): To a solution of **2** (100 mg, 0.14 mmol) in *o*-C_6_H_4_F_2_ (5 ml) was added AgBF_4_ (30 mg, 0.16 mmol). After stirring for 120 min at room temperature Cu(CF_3_Ac) (26 mg, 0.15 mmol) was added. The reaction mixture was stirred at room temperature overnight. The next day all volatiles were removed under high vacuum and the residue re-dissolved in CH_2_Cl_2_ (4 ml) and filtered over a syringe filter forming a yellow solution which was layered with *n*-hexane (5 ml). After 18 h the needle like crystalline product was collected, washed with *n*-pentane (4 × 3 ml) and dried under high vacuum at 70 °C for 2 d. Yield of **6** as a yellow crystalline solid 112 mg (85 %). Elemental Analysis for C_47_H_33_BCu_2_F_7_O_2_P (931.64 g/mol) found (calc.) [%]: C 60.60 (60.59), H 3.85 (3.57). ^1^H NMR (400 MHz, CD_2_Cl_2_, 25 °C): δ = 5.61 (d, ^2^*J*_P,H_ = 14.9 Hz, 3 H, H_benz_), 6.58 (s, 6 H, H_olef_), 6.94-6.97 (m, 6 H, H_aryl_), 7.12-7.19 (m, 12 H, H_aryl_), 7.23-7.27 (m, 6 H, H_aryl_) ppm. ^1^H NMR (400 MHz, CD_2_Cl_2_, −40 °C): δ = 5.54 (d, ^2^*J*_P,H_ = 13.8 Hz, 3 H, H_benz_), 6.56 (s, 6 H, H_olef_), 6.92-6.95 (m, 6 H, H_aryl_), 7.09-7.13 (m, 12 H, H_aryl_), 7.19-7.23 (m, 6 H, H_aryl_) ppm. ^13^C{^1^H} NMR (101 MHz, CD_2_Cl_2_, 25 °C): δ = 55.6 (d, ^1^*J*_P,C_ = 13.2 Hz, CH_aryl_), 120.0 (s, CH_olef_), 128.0 (s, CH_benz_), 129.8 (d, *J*_P,C_ = 1.8 Hz, CH_benz_), 131.6 (d, *J*_P,C_ = 4.9 Hz, CH_benz_), 131.7 (d, *J*_P,C_ = 2.8 Hz, C_benz_), 132.3 (s, CH_benz_), 134.5 (s, C_benz_) ppm. The ^13^C resonances of the TFA ligand could not be detected. ^13^C{^1^H} NMR (101 MHz, CD_2_Cl_2_, -40 °C): δ = 55.0 (d, ^1^*J*_P,C_ = 13.9 Hz, CH_aryl_), 119.2 (s, CH_olef_), 127.6 (s, CH_benz_), 129.3 (s, CH_benz_), 130.9 (d, *J*_P,C_ = 5.9 Hz, CH_benz_), 131.1 (d, *J*_P,C_ = 5.1 Hz, C_benz_), 131.8 (s, CH_benz_), 133.7 (s, C_benz_) ppm. The ^13^C resonances of the TFA ligand could not be detected. ^31^P{^1^H} NMR (162 MHz, CD_2_Cl_2_, 25 °C): δ = −33.4 (br, trop_3_P) ppm. ^31^P{^1^H} NMR (162 MHz, CD_2_Cl_2_, −40 °C): δ = −34.5 (br, trop_3_P) ppm. ^19^F{^1^H} NMR (376 MHz, CD_2_Cl_2_, 25 °C): δ = −73.4 (s, CF_3_), −148.6 (s, BF_4_) ppm. ^19^F{^1^H} NMR (376 MHz, CD_2_Cl_2_, −40 °C): δ = −73.1 (s, CF_3_), −148.0 (s, BF_4_) ppm.

# 2 NMR Spectra

Figure S1. ^1^H NMR spectrum of **1**.

Figure S2. ^13^C{^1^H} NMR spectrum of **1**.

Figure S3. ^31^P{^1^H} NMR spectrum of **1**.

Figure S4. ^1^H/^1^H COSY NMR spectrum of **1**.

Figure S5. ^1^H/^13^C{^1^H} HSQC NMR spectrum of **1**.

Figure S6. ^1^H/^13^C{^1^H} HMBC NMR spectrum of **1**.

Figure S7. ^1^H NMR spectrum of **2** at 25 °C.

Figure S8. ^1^H NMR spectrum of **2**.

Figure S9. ^13^C{^1^H} NMR spectrum of **2** at 25 °C.

Figure S10. ^31^P{^1^H} NMR spectrum of **2** at 25 °C.

Figure S11. ^31^P{^1^H} NMR spectrum of **2** at -40 °C.

Figure S12. ^1^H/^1^H COSY NMR spectrum of **2**.

Figure S13. ^1^H/^13^C{^1^H} HSQC NMR spectrum of **2**.

^13^C{^1^H}

^1^H

Figure S14. ^1^H/^13^C{^1^H} HMBC NMR spectrum of **2**.

Figure S15. ^1^H/^31^P{^1^H} HMBC NMR spectrum of **2**.

Figure S16. ^1^H NMR spectrum of **3** at 25 °C.

Figure S17. ^1^H NMR spectrum of **3** at −40 °C.

Figure S18. ^13^C{^1^H} NMR spectrum of **3** at 25 °C.

Figure S19. ^13^C APT NMR spectrum of **3** at 25 °C.

Figure S20. ^13^C APT NMR spectrum of **3** at −40 °C.

Figure S21. ^31^P{^1^H} NMR spectrum of **3** at 25 °C.

Figure S22. ^31^P{^1^H} NMR spectrum of **3** at −40 °C.

Figure S23. ^19^F{^1^H} NMR spectrum of **3** at 25 °C.

Figure S24. ^19^F{^1^H} NMR spectrum of **3** at −40 °C.

Figure S25. ^1^H/^1^H COSY NMR spectrum of **3** at 27 °C.

Figure S26. ^1^H/^13^C{^1^H} HSQC NMR spectrum of **3** at 27 °C.

Figure S27. ^1^H/^13^C{^1^H} HMBC NMR spectrum of **3** at 27 °C.

Figure S28. ^1^H NMR spectrum of **4** at 25 °C.

Figure S29. ^1^H NMR spectrum of **4** at −40 °C.

Figure S30. ^31^P{^1^H} NMR spectrum of **4** at 25 °C.

Figure S31. ^31^P{^1^H} NMR spectrum of **4** at −40 °C.

Figure S32. ^19^F{^1^H} NMR spectrum of **4** at 25 °C.

Figure S33. ^19^F{^1^H} NMR spectrum of **4** at −40 °C.

Figure S34. ^13^C APT NMR spectrum of **4** at −40 °C.

Figure S35. ^1^H/^1^H NOESY-EXSY NMR spectra of **4** at −40 °C.

Figure S36. HSQC NMR spectra of **4** at −40 °C.

Figure S37. ^1^H/^31^P{^1^H} HMBC NMR of **4** at −40 °C.

Figure S38. ^1^H NMR of **4’** at 28 °C.

Figure S39. ^1^H NMR of **4’** at −40 °C.

Figure S40. ^13^C{^1^H} NMR of **4’** at 28 °C.

Figure S41. ^13^C APT of **4’** at 28 °C.

Figure S42. ^31^P{^1^H} NMR spectrum of **4’** at 28 °C.

Figure S43. ^31^P{^1^H} NMR spectrum of **4’** at −40 °C.

Figure S44. ^19^F{^1^H} NMR spectrum of **4’** at −40 °C.

Figure S45. ^1^H/^13^C HSQC NMR spectrum of **4’** at 27 °C.

Figure S46. ^1^H NMR spectrum of **5** at 26 °C.

Figure S47. ^1^H NMR spectrum of **5** at −40 °C.

Figure S48. ^13^C{^1^H} NMR spectrum of **5** at −40 °C.

Figure S49. ^31^P{^1^H} NMR spectrum of **5** at 25 °C.

Figure S50. ^31^P{^1^H} NMR spectrum of **5** at −40 °C.

Figure S51. ^19^F{^1^H} NMR spectrum of **5** at 25 °C.

Figure S52. ^19^F{^1^H} NMR spectrum of **5** at −40 °C.

Figure S53. ^1^H/^13^C{^1^H} HSQC NMR of **5** at −40 °C.

Figure S54. ^1^H/^31^P{^1^H} HMBC NMR of **5** at −40 °C.

Figure S55. ^1^H/^1^H NOESY-EXCY NMR of **5** at −40 °C.

Figure S56. ^1^H NMR spectrum of **6** at 25 °C.

Figure S57. ^1^H NMR spectrum of **6** at −40 °C.

Figure S58. ^13^C{^1^H} NMR spectrum of **6** at 25 °C.

Figure S59. ^13^C APT NMR spectrum of **6** at −40 °C.

Figure S60. ^31^P{^1^H} NMR spectrum of **6** at 25 °C.

Figure S61. ^31^P{^1^H} NMR spectrum of **6** at −40 °C.

Figure S62. ^19^F{^1^H} NMR spectrum of **6** at 25 °C.

Figure S63. ^19^F{^1^H} NMR spectrum of **6** at −40 °C.

Figure S64. ^1^H/^1^H COSY NMR spectrum of **6** at 27 °C.

Figure S65. ^1^H/^13^C{^1^H} HSQC NMR spectrum of **6** at 25 °C.

Figure S66. ^1^H/^13^C{^1^H} HMBC NMR spectrum of **6** at 27 °C.

## 2.1 VT-NMR Experiments

Figure S67. ^1^H NMR spectrum of **4** at variable temperature in CD_2_Cl_2_.

Figure S68. ^1^H NMR spectrum of **5** at variable temperature in CD_2_Cl_2_.

Figure S69. Equilibrium of complex **4** with compound **4’**. Selected parts of ^1^H NMR spectra of **4** at different temperatures.

Figure S70. Eyring and Arrhenius plot of complex **4**.

ln(k/T)

ln(k)

| 1/T [K-1] | Earing |
| --- | --- |
| 0.0033557 | -1.93589 |
| 0.00359712 | -2.36952 |
| 0.00387597 | -3.25037 |
| 0.00429185 | -4.66258 |

|  | Ahrenius |
| --- | --- |
| 0.0033557 | 3.7612 |
| 0.00359712 | 3.2581 |
| 0.00387597 | 2.30259 |
| 0.00429185 | 0.788457 |

Figure S71. Eyring and Arrhenius plot of complex **5**.

ln(k/T)

ln(K)

| 1/T [K-1] | Earing |
| --- | --- |
| 0.0033557 | -0.834185 |
| 0.00359712 | -3.07039 |
| 0.00387597 | -4.07136 |
| 0.00429185 | -6.68891 |

|  | Ahrenius |
| --- | --- |
| 0.0033557 | 4.86291 |
| 0.00359712 | 2.55723 |
| 0.00387597 | 1.4816 |
| 0.00429185 | -1.23787 |

# 3 MS Spectra

Figure S72. ESI-MS spectra positive ion of **2**.

Figure S73. ESI-MS spectra positive ion of **3**.

Figure S74. ESI-MS spectra positive ion of **4**.

Figure S75. ESI-MS spectra positive ion of **4’**.

Figure S76. ESI-MS spectra positive ion of **5**.

Figure S77. ESI-MS spectra positive ion of **6**.

# 4 IR-ATR Spectra

Figure S78. IR-ATR spectra of **2**.

Figure S79. IR-ATR spectra of **3**.

Figure S80. IR-ATR spectra of **5**.

# 5 UV-VIS Spectra

Figure S81. UV-Vis spectra of complex **2**,**3**,**4**,**4’**,**5** and **6** recorded in CH_2_Cl_2_ at room temperature.

# 6 Crystallographic Data

Complex **2:**

Figure S2: Molecular structure of complex **2** with displacement ellipsoids drawn at 50 % probability level. H atoms and a co-crystallized dichloromethane solvent molecules are omitted for clarity (C in black, P in pink, Cu in blue, Cl in green).

| Table S1: Crystallographic data for complex [Cu(trop_3_P)Cl] (**2**). | |
| --- | --- |
| Identification code | dh_cu046_4_auto |
| Deposition number | CCDC-2501174 |
| Empirical formula | C_46_H_35_Cl_3_CuP |
| Formula weight | 788.60 |
| Temperature/K | 100.00(10) |
| Crystal system | monoclinic |
| Space group | *Cc* |
| a/Å | 14.70910(10) |
| b/Å | 19.0479(2) |
| c/Å | 13.43600(10) |
| α/° | 90 |
| β/° | 102.1260(10) |
| γ/° | 90 |
| Volume/Å^3^ | 3680.47(6) |
| Z | 4 |
| ρ_calc_g/cm^3^ | 1.423 |
| μ/mm^‑1^ | 3.503 |
| F(000) | 1624.0 |
| Crystal size/mm^3^ | 0.123 × 0.087 × 0.065 |
| Radiation | Cu Kα (λ = 1.54184) |
| 2Θ range for data collection/° | 7.702 to 160.278 |
| Index ranges | -14 ≤ h ≤ 18, -21 ≤ k ≤ 24, -16 ≤ l ≤ 17 |
| Reflections collected | 16625 |
| Independent reflections | 5423 [R_int_ = 0.0284, R_sigma_ = 0.0302] |
| Data/restraints/parameters | 5423/2/460 |
| Goodness-of-fit on F^2^ | 1.045 |
| Final R indexes [I>=2σ (I)] | R_1_ = 0.0266, wR_2_ = 0.0675 |
| Final R indexes [all data] | R_1_ = 0.0272, wR_2_ = 0.0679 |
| Largest diff. peak/hole / e Å^-3^ | 0.33/-0.35 |
| Flack parameter | 0.000(11) |

| Table S2: Interatomic distances for complex [Cu(trop_3_P)Cl] (**2**). | | | | | | |
| --- | --- | --- | --- | --- | --- | --- |
| **Atom** | **Atom** | **Length/Å** |  | **Atom** | **Atom** | **Length/Å** |
| Cu1 | Cl1 | 2.1604(8) |  | C21 | C22 | 1.411(4) |
| Cu1 | P1 | 2.1756(8) |  | C22 | C23 | 1.460(4) |
| P1 | C1 | 1.887(3) |  | C23 | C24 | 1.351(4) |
| P1 | C16 | 1.884(3) |  | C24 | C25 | 1.471(4) |
| P1 | C31 | 1.910(3) |  | C25 | C26 | 1.401(4) |
| C1 | C2 | 1.515(4) |  | C25 | C30 | 1.408(4) |
| C1 | C15 | 1.519(4) |  | C26 | C27 | 1.383(5) |
| C2 | C3 | 1.401(4) |  | C27 | C28 | 1.387(5) |
| C2 | C7 | 1.407(4) |  | C28 | C29 | 1.390(4) |
| C3 | C4 | 1.388(4) |  | C29 | C30 | 1.404(4) |
| C4 | C5 | 1.390(4) |  | C31 | C32 | 1.514(4) |
| C5 | C6 | 1.387(4) |  | C31 | C45 | 1.514(4) |
| C6 | C7 | 1.408(4) |  | C32 | C33 | 1.398(4) |
| C7 | C8 | 1.469(4) |  | C32 | C37 | 1.402(4) |
| C8 | C9 | 1.341(4) |  | C33 | C34 | 1.390(4) |
| C9 | C10 | 1.465(4) |  | C34 | C35 | 1.385(5) |
| C10 | C11 | 1.407(4) |  | C35 | C36 | 1.388(4) |
| C10 | C15 | 1.404(4) |  | C36 | C37 | 1.411(4) |
| C11 | C12 | 1.383(5) |  | C37 | C38 | 1.463(4) |
| C12 | C13 | 1.390(5) |  | C38 | C39 | 1.344(4) |
| C13 | C14 | 1.392(4) |  | C39 | C40 | 1.461(4) |
| C14 | C15 | 1.397(4) |  | C40 | C41 | 1.409(4) |
| C16 | C17 | 1.517(4) |  | C40 | C45 | 1.410(4) |
| C16 | C30 | 1.521(4) |  | C41 | C42 | 1.377(5) |
| C17 | C18 | 1.395(4) |  | C42 | C43 | 1.393(4) |
| C17 | C22 | 1.400(4) |  | C43 | C44 | 1.392(4) |
| C18 | C19 | 1.392(4) |  | C44 | C45 | 1.394(4) |
| C19 | C20 | 1.396(5) |  | Cl2 | C46 | 1.761(5) |
| C20 | C21 | 1.380(5) |  | Cl3 | C46 | 1.761(4) |

 Complex **3:**

Figure S3: Molecular structure of complex **3** (molecule one of the two crystallographically independent molecules in the asymmetric unit) with displacement ellipsoids drawn at 50 % probability level. H atoms are omitted for clarity (C in black, P in pink, Cu in blue, O in red, S in yellow, F in green).

Figure S4: Molecular structure of complex **3** (molecule two of the two crystallographically independent molecules in the asymmetric unit) with displacement ellipsoids drawn at 50 % probability level. H atoms are omitted for clarity. The component with minor occupancy (36 %) of a disordered CF_3_-group is shown in turquoise (C in black, P in pink, Cu in blue, O in red, S in yellow, F in green).

Refinement details: The F-atoms of one of the triflate groups are disordered over two sites and are described with split positions. For the F-F-distances and C-F-bonds, distance restraints (SADI) were used as well as motion constraints (EADP) for C and motion restraints (RIGU) with smaller than default esds for the whole anion. A residual density of about 2 e-/A3 most likely originates from a disorder of the chains, which are formed by stacking the molecules along the a-c diagonal.

| Table S3: Crystallographic data for complex [Cu(trop_3_P)(OTf)] (**3**),. | |
| --- | --- |
| Identification code | dh_cu062_2 |
| Deposition number | CCDC-2501175 |
| Empirical formula | C_46_H_33_CuF_3_O_3_PS |
| Formula weight | 817.29 |
| Temperature/K | 100.0(2) |
| Crystal system | monoclinic |
| Space group | *Pn* |
| a/Å | 13.2325(2) |
| b/Å | 18.1537(2) |
| c/Å | 15.6830(2) |
| α/° | 90 |
| β/° | 101.6540(10) |
| γ/° | 90 |
| Volume/Å^3^ | 3689.69(8) |
| Z | 4 |
| ρ_calc_g/cm^3^ | 1.471 |
| μ/mm^‑1^ | 2.264 |
| F(000) | 1680.0 |
| Crystal size/mm^3^ | 0.13 × 0.09 × 0.05 |
| Radiation | Cu Kα (λ = 1.54184) |
| 2Θ range for data collection/° | 4.868 to 160.738 |
| Index ranges | -16 ≤ h ≤ 16, -23 ≤ k ≤ 20, -20 ≤ l ≤ 19 |
| Reflections collected | 30394 |
| Independent reflections | 12458 [R_int_ = 0.0357, R_sigma_ = 0.0434] |
| Data/restraints/parameters | 12458/123/1022 |
| Goodness-of-fit on F^2^ | 1.040 |
| Final R indexes [I>=2σ (I)] | R_1_ = 0.0641, wR_2_ = 0.1732 |
| Final R indexes [all data] | R_1_ = 0.0677, wR_2_ = 0.1755 |
| Largest diff. peak/hole / e Å^-3^ | 2.25/-0.67 |
| Flack parameter | 0.029(11) |

| Table S4 Crystallographic data for complex [Cu(trop_3_P)(OTf)] (**3**). | | | | | | |
| --- | --- | --- | --- | --- | --- | --- |
| **Atom** | **Atom** | **Length/Å** |  | **Atom** | **Atom** | **Length/Å** |
| Cu2 | P2 | 2.1634(19) |  | F5 | C92 | 1.314(15) |
| Cu2 | O4 | 1.974(5) |  | F6 | C92 | 1.340(14) |
| S1 | O4 | 1.432(6) |  | Cu1 | P1 | 2.170(2) |
| S1 | O5 | 1.426(6) |  | Cu1 | O1 | 1.959(6) |
| S1 | O6 | 1.435(6) |  | S2 | O1 | 1.440(6) |
| S1 | C92A | 1.85(3) |  | S2 | O2 | 1.419(6) |
| S1 | C92 | 1.784(19) |  | S2 | O3 | 1.436(6) |
| P2 | C46 | 1.877(7) |  | S2 | C91 | 1.836(9) |
| P2 | C61 | 1.886(7) |  | P1 | C1 | 1.877(8) |
| P2 | C76 | 1.887(7) |  | P1 | C16 | 1.892(7) |
| C46 | C47 | 1.520(10) |  | P1 | C31 | 1.883(7) |
| C46 | C60 | 1.526(9) |  | F1 | C91 | 1.315(10) |
| C47 | C48 | 1.402(11) |  | F2 | C91 | 1.317(10) |
| C47 | C52 | 1.388(11) |  | F3 | C91 | 1.337(11) |
| C48 | C49 | 1.373(12) |  | C1 | C2 | 1.517(10) |
| C49 | C50 | 1.405(14) |  | C1 | C15 | 1.526(10) |
| C50 | C51 | 1.365(14) |  | C2 | C3 | 1.397(11) |
| C51 | C52 | 1.406(12) |  | C2 | C7 | 1.396(11) |
| C52 | C53 | 1.465(12) |  | C3 | C4 | 1.361(12) |
| C53 | C54 | 1.361(12) |  | C4 | C5 | 1.385(13) |
| C54 | C55 | 1.469(12) |  | C5 | C6 | 1.373(12) |
| C55 | C56 | 1.392(11) |  | C6 | C7 | 1.396(11) |
| C55 | C60 | 1.414(10) |  | C7 | C8 | 1.475(11) |
| C56 | C57 | 1.383(14) |  | C8 | C9 | 1.345(13) |
| C57 | C58 | 1.394(14) |  | C9 | C10 | 1.449(13) |
| C58 | C59 | 1.391(11) |  | C10 | C11 | 1.414(12) |
| C59 | C60 | 1.387(10) |  | C10 | C15 | 1.411(12) |
| C61 | C62 | 1.498(10) |  | C11 | C12 | 1.374(16) |
| C61 | C75 | 1.510(9) |  | C12 | C13 | 1.350(17) |
| C62 | C63 | 1.409(11) |  | C13 | C14 | 1.393(14) |
| C62 | C67 | 1.403(11) |  | C14 | C15 | 1.382(12) |
| C63 | C64 | 1.380(12) |  | C16 | C17 | 1.534(10) |
| C64 | C65 | 1.423(15) |  | C16 | C30 | 1.529(10) |
| C65 | C66 | 1.346(14) |  | C17 | C18 | 1.379(12) |
| C66 | C67 | 1.414(11) |  | C17 | C22 | 1.418(11) |
| C67 | C68 | 1.476(12) |  | C18 | C19 | 1.393(13) |
| C68 | C69 | 1.338(12) |  | C19 | C20 | 1.336(14) |
| C69 | C70 | 1.475(11) |  | C20 | C21 | 1.369(13) |
| C70 | C71 | 1.391(11) |  | C21 | C22 | 1.415(11) |
| C70 | C75 | 1.412(10) |  | C22 | C23 | 1.452(11) |
| C71 | C72 | 1.416(12) |  | C23 | C24 | 1.372(12) |
| C72 | C73 | 1.370(12) |  | C24 | C25 | 1.452(11) |
| C73 | C74 | 1.386(11) |  | C25 | C26 | 1.426(11) |
| C74 | C75 | 1.394(10) |  | C25 | C30 | 1.416(11) |
| C76 | C77 | 1.506(9) |  | C26 | C27 | 1.374(13) |
| C76 | C90 | 1.504(10) |  | C27 | C28 | 1.389(12) |
| C77 | C78 | 1.388(10) |  | C28 | C29 | 1.374(11) |
| C77 | C82 | 1.394(10) |  | C29 | C30 | 1.357(11) |
| C78 | C79 | 1.388(10) |  | C31 | C32 | 1.528(10) |
| C79 | C80 | 1.389(13) |  | C31 | C45 | 1.523(10) |
| C80 | C81 | 1.404(12) |  | C32 | C33 | 1.386(11) |
| C81 | C82 | 1.410(10) |  | C32 | C37 | 1.406(11) |
| C82 | C83 | 1.481(11) |  | C33 | C34 | 1.378(12) |
| C83 | C84 | 1.330(12) |  | C34 | C35 | 1.364(13) |
| C84 | C85 | 1.469(11) |  | C35 | C36 | 1.377(13) |
| C85 | C86 | 1.400(12) |  | C36 | C37 | 1.421(12) |
| C85 | C90 | 1.405(10) |  | C37 | C38 | 1.462(12) |
| C86 | C87 | 1.374(14) |  | C38 | C39 | 1.337(12) |
| C87 | C88 | 1.376(14) |  | C39 | C40 | 1.480(11) |
| C88 | C89 | 1.384(12) |  | C40 | C41 | 1.420(11) |
| C89 | C90 | 1.379(11) |  | C40 | C45 | 1.367(11) |
| F4A | C92A | 1.312(18) |  | C41 | C42 | 1.368(12) |
| F5A | C92A | 1.315(18) |  | C42 | C43 | 1.375(13) |
| F6A | C92A | 1.336(18) |  | C43 | C44 | 1.398(10) |
| F4 | C92 | 1.322(15) |  | C44 | C45 | 1.407(10) |

Complex **4:**

Figure S5: Molecular structure of complex **4** with displacement ellipsoids drawn at 50 % probability level. H atoms, a disordered BF_4_^-^-anion as well as the component of the disordered complex molecule with minor occupancy (17 %) are omitted for clarity. (C in black, P in pink, Cu in blue, Cl in green).

Refinement details: The complete cation is disordered over two sites with an occupancy ratio of 83:17. While the site with larger occupancy (PART 1) could be refined without the need of distance or geometric restraints, the site with only 17 percent occupancy (PART 2) had to be refined using many distance and geometry restraints and constraints (SADI, AFIX 66, FLAT). Also, anisotropic displacement parameters of this minor component had to be restraint using a combination of RIGU, and ISOR with smaller than default values for the corresponding esds in order to obtain a stable refinement. A RIGU restraint with smaller than default values has been used for the main component of the cation, too. Despite some further fairly anisotropic ellipsoids for the main site with 83 percent occupancy, we decided to not introduce additional split sites in order to avoid further complications by introducing even more parameters, as the current refinement has already a data/parameter ratio of close to 8. The BF_4_^-^-anion is disordered over three sites. Two positions (PART 1 and 3) with an occupancy ratio of 0.46:0.371 which belong to the main component of the cation, were refined with anisotropic displacement parameters, using distance (SADI) and displacement restraints (RIGU, ISOR, EADP) with smaller than default esds. The third position (PART 2), which belongs to the minor component of the cation (both occupancy factors constraint to be equal at 17 %) has been refined using a rigid group description (AFIX 6) and isotropic displacement parameters. There are indications that the crystal under investigation features a modulated structure. It is possible to index the reflections also with a smaller unit cell with monoclinic lattice constants of a = 13.1774(7) Å, b = 10.3226(4) Å, c = 13.1739(6) Å, beta = 95.424(4)°, cell volume 1793.95(15) Å^3^and a modulation vector (0.241 0 0.241) with only first order satellites. However, all attempts to solve and refine the structure in a 3+1D space group were unsuccessful. Still, this might explain the somewhat unsatisfactory structural description as an approximant, using a conventional 3D space group and a 4 times larger unit cell.

| Table S5: Crystallographic data for complex [Cu_2_(trop_3_P)(μ^2^-Cl)]BF_4_ (**4**). | |
| --- | --- |
| Identification code | dh_cu070_1_fromdcm_on |
| Deposition number | CCDC-2501180 |
| Empirical formula | C_45_H_33_BClCu_2_F_4_P |
| Formula weight | 854.02 |
| Temperature/K | 100.01(11) |
| Crystal system | orthorhombic |
| Space group | *Pbca* |
| a/Å | 19.4998(4) |
| b/Å | 10.32533(14) |
| c/Å | 35.4273(7) |
| α/° | 90 |
| β/° | 90 |
| γ/° | 90 |
| Volume/Å^3^ | 7133.0(2) |
| Z | 8 |
| ρ_calc_g/cm^3^ | 1.591 |
| μ/mm^‑1^ | 3.046 |
| F(000) | 3472.0 |
| Crystal size/mm^3^ | 0.065 × 0.048 × 0.019 |
| Radiation | Cu Kα (λ = 1.54184) |
| 2Θ range for data collection/° | 4.988 to 160.742 |
| Index ranges | -23 ≤ h ≤ 24, -13 ≤ k ≤ 5, -44 ≤ l ≤ 44 |
| Reflections collected | 32546 |
| Independent reflections | 7447 [R_int_ = 0.0449, R_sigma_ = 0.0385] |
| Data/restraints/parameters | 7447/1245/909 |
| Goodness-of-fit on F^2^ | 1.115 |
| Final R indexes [I>=2σ (I)] | R_1_ = 0.0937, wR_2_ = 0.2342 |
| Final R indexes [all data] | R_1_ = 0.1105, wR_2_ = 0.2426 |
| Largest diff. peak/hole / e Å^-3^ | 0.52/-1.12 |

| Table S6: Interatomic distances for complex [Cu_2_(trop_3_P)(μ^2^-Cl)]BF_4_ (**4**). | | | | | | |
| --- | --- | --- | --- | --- | --- | --- |
| **Atom** | **Atom** | **Length/Å** |  | **Atom** | **Atom** | **Length/Å** |
| Cu1 | Cu2 | 2.4401(13) |  | Cu1A | P1A | 2.221(14) |
| Cu1 | Cl1 | 2.2225(19) |  | Cu2A | Cl1A | 2.354(14) |
| Cu1 | P1 | 2.2592(19) |  | Cu2A | P1A | 2.588(19) |
| Cu2 | Cl1 | 2.3392(18) |  | P1A | C1A | 1.88(5) |
| Cu2 | P1 | 2.645(2) |  | P1A | C31A | 1.96(5) |
| P1 | C1 | 1.897(7) |  | P1A | C16A | 1.85(6) |
| P1 | C16 | 1.889(7) |  | F4 | B1 | 1.369(14) |
| P1 | C31 | 1.903(8) |  | F2 | B1 | 1.373(13) |
| C1 | C2 | 1.516(11) |  | F3 | B1 | 1.357(13) |
| C1 | C15 | 1.517(11) |  | F1 | B1 | 1.369(13) |
| C2 | C3 | 1.392(12) |  | C1A | C2A | 1.52(3) |
| C2 | C7 | 1.414(12) |  | C1A | C15A | 1.52(3) |
| C3 | C4 | 1.432(15) |  | C2A | C3A | 1.3900 |
| C4 | C5 | 1.367(18) |  | C2A | C7A | 1.3900 |
| C5 | C6 | 1.379(16) |  | C3A | C4A | 1.3900 |
| C6 | C7 | 1.392(12) |  | C4A | C5A | 1.3900 |
| C7 | C8 | 1.459(12) |  | C5A | C6A | 1.3900 |
| C8 | C9 | 1.367(11) |  | C6A | C7A | 1.3900 |
| C9 | C10 | 1.460(10) |  | C7A | C8A | 1.43(3) |
| C10 | C11 | 1.412(11) |  | C38A | C37A | 1.43(3) |
| C10 | C15 | 1.403(11) |  | C38A | C39A | 1.31(5) |
| C11 | C12 | 1.388(12) |  | C23A | C24A | 1.31(5) |
| C12 | C13 | 1.399(14) |  | C23A | C22A | 1.44(3) |
| C13 | C14 | 1.379(13) |  | C31A | C32A | 1.52(3) |
| C14 | C15 | 1.387(11) |  | C31A | C45A | 1.53(3) |
| C16 | C17 | 1.519(12) |  | C8A | C9A | 1.33(5) |
| C16 | C30 | 1.550(11) |  | C9A | C10A | 1.44(3) |
| C17 | C18 | 1.390(13) |  | C24A | C25A | 1.44(3) |
| C17 | C22 | 1.417(13) |  | C30A | C29A | 1.3900 |
| C18 | C19 | 1.359(15) |  | C30A | C25A | 1.3900 |
| C19 | C20 | 1.332(18) |  | C30A | C16A | 1.52(3) |
| C20 | C21 | 1.416(16) |  | C29A | C28A | 1.3900 |
| C21 | C22 | 1.413(13) |  | C28A | C27A | 1.3900 |
| C22 | C23 | 1.453(12) |  | C27A | C26A | 1.3900 |
| C23 | C24 | 1.358(11) |  | C26A | C25A | 1.3900 |
| C24 | C25 | 1.452(12) |  | C15A | C10A | 1.3900 |
| C25 | C26 | 1.414(11) |  | C15A | C14A | 1.3900 |
| C25 | C30 | 1.385(12) |  | C10A | C11A | 1.3900 |
| C26 | C27 | 1.385(15) |  | C11A | C12A | 1.3900 |
| C27 | C28 | 1.339(17) |  | C12A | C13A | 1.3900 |
| C28 | C29 | 1.419(14) |  | C13A | C14A | 1.3900 |
| C29 | C30 | 1.360(13) |  | C32A | C33A | 1.3900 |
| C31 | C32 | 1.500(11) |  | C32A | C37A | 1.3900 |
| C31 | C45 | 1.506(12) |  | C33A | C34A | 1.3900 |
| C32 | C33 | 1.411(12) |  | C34A | C35A | 1.3900 |
| C32 | C37 | 1.380(12) |  | C35A | C36A | 1.3900 |
| C33 | C34 | 1.384(16) |  | C36A | C37A | 1.3900 |
| C34 | C35 | 1.388(19) |  | C16A | C17A | 1.52(3) |
| C35 | C36 | 1.368(14) |  | C17A | C22A | 1.3900 |
| C36 | C37 | 1.403(11) |  | C17A | C18A | 1.3900 |
| C37 | C38 | 1.460(10) |  | C22A | C21A | 1.3900 |
| C38 | C39 | 1.371(10) |  | C21A | C20A | 1.3900 |
| C39 | C40 | 1.456(12) |  | C20A | C19A | 1.3900 |
| C40 | C41 | 1.423(12) |  | C19A | C18A | 1.3900 |
| C40 | C45 | 1.396(12) |  | C39A | C40A | 1.43(3) |
| C41 | C42 | 1.363(15) |  | C45A | C44A | 1.3900 |
| C42 | C43 | 1.368(18) |  | C45A | C40A | 1.3900 |
| C43 | C44 | 1.386(16) |  | C44A | C43A | 1.3900 |
| C44 | C45 | 1.418(13) |  | C43A | C42A | 1.3900 |
| F5 | B2 | 1.378(13) |  | C42A | C41A | 1.3900 |
| F6 | B2 | 1.370(12) |  | C41A | C40A | 1.3900 |
| F7 | B2 | 1.366(12) |  | B3 | F12 | 1.4185 |
| F8 | B2 | 1.370(12) |  | B3 | F10 | 1.4185 |
| Cu1A | Cu2A | 2.452(9) |  | B3 | F11 | 1.4186 |
| Cu1A | Cl1A | 2.242(13) |  | B3 | F9 | 1.4187 |

Complex **4’:**

Figure S6: Molecular structure of complex **4’** (molecule one of the two crystallographically independent molecules in the asymmetric unit) with displacement ellipsoids drawn at 50 % probability level. H atoms and a co-crystallized dichloromethane solvent molecule are omitted for clarity (C in black, P in pink, Cu in blue, F in green, B in yellow).

Figure S7: Molecular structure of complex **4’** (molecule two of the two crystallographically independent molecules in the asymmetric unit) with displacement ellipsoids drawn at 50 % probability level. H atoms and a co-crystallized dichloromethane solvent molecule are omitted for clarity (C in black, P in pink, Cu in blue, F in green, B in yellow).

Refinement details: A masked refinement was applied for an area that contains dichloromethane and most likely hexane (in total corresponding to 2 dichloromethane molecules per asymmetric unit), because all attempts to unambiguously resolve the disorder failed. One dichloromethane molecule is disordered over two positions around the mirror plane perpendicular to the crystallographic b-axis.

| Table S7: Crystallographic data for complex [Cu_2_(trop_3_P)(BF_4_] (**4’**). | |
| --- | --- |
| Identification code | dh_cu070_4_supernat_auto |
| Deposition number | CCDC-2501177 |
| Empirical formula | C_136_H_101_B_3_Cl_2_Cu_3_F_12_P_3_ |
| Formula weight | 2350.02 |
| Temperature/K | 100.03(13) |
| Crystal system | orthorhombic |
| Space group | *Pnma* |
| a/Å | 18.20520(14) |
| b/Å | 50.5116(4) |
| c/Å | 12.38737(8) |
| α/° | 90 |
| β/° | 90 |
| γ/° | 90 |
| Volume/Å^3^ | 11391.10(14) |
| Z | 4 |
| ρ_calc_g/cm^3^ | 1.370 |
| μ/mm^‑1^ | 2.069 |
| F(000) | 4824.0 |
| Crystal size/mm^3^ | 0.118 × 0.087 × 0.065 |
| Radiation | Cu Kα (λ = 1.54184) |
| 2Θ range for data collection/° | 8.634 to 161.244 |
| Index ranges | -23 ≤ h ≤ 22, -63 ≤ k ≤ 64, -15 ≤ l ≤ 13 |
| Reflections collected | 130443 |
| Independent reflections | 12544 [R_int_ = 0.0703, R_sigma_ = 0.0342] |
| Data/restraints/parameters | 12544/0/740 |
| Goodness-of-fit on F^2^ | 1.170 |
| Final R indexes [I>=2σ (I)] | R_1_ = 0.0716, wR_2_ = 0.1655 |
| Final R indexes [all data] | R_1_ = 0.0745, wR_2_ = 0.1671 |
| Largest diff. peak/hole / e Å^-3^ | 0.98/-0.56 |

| Table S8: Interatomic distances for complex [Cu_2_(trop_3_P)(BF_4_] (**4’**). | | | | | | |
| --- | --- | --- | --- | --- | --- | --- |
| **Atom** | **Atom** | **Length/Å** |  | **Atom** | **Atom** | **Length/Å** |
| Cu1 | P1 | 2.1835(9) |  | C36 | C37 | 1.402(5) |
| Cu1 | F1 | 2.107(2) |  | C37 | C38 | 1.464(5) |
| P1 | C1 | 1.879(4) |  | C38 | C39 | 1.349(6) |
| P1 | C16 | 1.876(3) |  | C39 | C40 | 1.474(5) |
| P1 | C31 | 1.881(3) |  | C40 | C41 | 1.392(6) |
| F1 | B1 | 1.426(5) |  | C40 | C45 | 1.408(5) |
| F2 | B1 | 1.392(6) |  | C41 | C42 | 1.388(6) |
| F3 | B1 | 1.370(6) |  | C42 | C43 | 1.380(7) |
| F4 | B1 | 1.363(5) |  | C43 | C44 | 1.382(6) |
| C1 | C2 | 1.522(5) |  | C44 | C45 | 1.407(5) |
| C1 | C15 | 1.514(5) |  | Cu2 | P2 | 2.1988(12) |
| C2 | C3 | 1.390(5) |  | Cu2 | F5 | 2.113(3) |
| C2 | C7 | 1.415(5) |  | Cu2 | C53^1^ | 2.260(4) |
| C3 | C4 | 1.375(6) |  | Cu2 | C54^1^ | 2.306(4) |
| C4 | C5 | 1.403(6) |  | P2 | C46^1^ | 1.877(3) |
| C5 | C6 | 1.371(6) |  | P2 | C46 | 1.877(3) |
| C6 | C7 | 1.399(6) |  | P2 | C61 | 1.883(5) |
| C7 | C8 | 1.470(5) |  | F5 | B2 | 1.406(6) |
| C8 | C9 | 1.360(6) |  | F6 | B2 | 1.382(4) |
| C9 | C10 | 1.457(6) |  | F7 | B2 | 1.388(6) |
| C10 | C11 | 1.406(6) |  | C46 | C47 | 1.514(4) |
| C10 | C15 | 1.407(6) |  | C46 | C60 | 1.529(4) |
| C11 | C12 | 1.374(8) |  | C47 | C48 | 1.390(5) |
| C12 | C13 | 1.385(9) |  | C47 | C52 | 1.400(5) |
| C13 | C14 | 1.387(6) |  | C48 | C49 | 1.404(6) |
| C14 | C15 | 1.394(6) |  | C49 | C50 | 1.378(9) |
| C16 | C17 | 1.532(5) |  | C50 | C51 | 1.383(8) |
| C16 | C30 | 1.509(5) |  | C51 | C52 | 1.407(6) |
| C17 | C18 | 1.390(5) |  | C52 | C53 | 1.482(6) |
| C17 | C22 | 1.400(6) |  | C53 | C54 | 1.343(6) |
| C18 | C19 | 1.393(6) |  | C54 | C55 | 1.459(6) |
| C19 | C20 | 1.373(8) |  | C55 | C56 | 1.413(5) |
| C20 | C21 | 1.384(7) |  | C55 | C60 | 1.408(5) |
| C21 | C22 | 1.401(5) |  | C56 | C57 | 1.367(8) |
| C22 | C23 | 1.469(5) |  | C57 | C58 | 1.390(8) |
| C23 | C24 | 1.351(5) |  | C58 | C59 | 1.388(6) |
| C24 | C25 | 1.461(6) |  | C59 | C60 | 1.382(5) |
| C25 | C26 | 1.394(5) |  | C61 | C62 | 1.520(4) |
| C25 | C30 | 1.416(5) |  | C61 | C62^1^ | 1.520(4) |
| C26 | C27 | 1.389(7) |  | C62 | C63 | 1.385(5) |
| C27 | C28 | 1.374(7) |  | C62 | C67 | 1.402(5) |
| C28 | C29 | 1.399(6) |  | C63 | C64 | 1.408(7) |
| C29 | C30 | 1.388(6) |  | C64 | C65 | 1.400(9) |
| C31 | C32 | 1.518(5) |  | C65 | C66 | 1.360(8) |
| C31 | C45 | 1.511(5) |  | C66 | C67 | 1.406(5) |
| C32 | C33 | 1.395(5) |  | C67 | C68 | 1.468(5) |
| C32 | C37 | 1.402(5) |  | C68 | C68^1^ | 1.326(8) |
| C33 | C34 | 1.379(6) |  | Cl1 | C69 | 1.756(9) |
| C34 | C35 | 1.395(7) |  | Cl2 | C69 | 1.768(8) |
| C35 | C36 | 1.386(6) |  |  |  |  |

^1^+X,3/2-Y,+Z

Complex **5:**

Figure S8: Molecular structure of complex **5** with displacement ellipsoids drawn at 50 % probability level. H atoms, the counter anion BF_4_^-^ and a co-crystallized dichloromethane solvent molecule are omitted for clarity (C in black, P in pink, Cu in blue, O in red, S in yellow, F in green).

Refinement details: The six H-atoms at the olefinic C=C_trop_ binding site were located in the difference Fourier map and refined freely.

| Table S9: Crystallographic data for complex [Cu_2_(trop_3_P)(TFA)]BF_4_ (**5**). | |
| --- | --- |
| Identification code | dh_cu057_2_auto |
| Deposition number | CCDC-2501179 |
| Empirical formula | C_47_H_35_BCl_2_Cu_2_F_7_O_3_PS |
| Formula weight | 1052.57 |
| Temperature/K | 100.0(4) |
| Crystal system | monoclinic |
| Space group | *P*2_1_/*c* |
| a/Å | 18.16410(10) |
| b/Å | 10.36250(10) |
| c/Å | 23.8378(2) |
| α/° | 90 |
| β/° | 108.5600(10) |
| γ/° | 90 |
| Volume/Å^3^ | 4253.52(6) |
| Z | 4 |
| ρ_calc_g/cm^3^ | 1.644 |
| μ/mm^‑1^ | 3.852 |
| F(000) | 2128.0 |
| Crystal size/mm^3^ | 0.093 × 0.085 × 0.02 |
| Radiation | Cu Kα (λ = 1.54184) |
| 2Θ range for data collection/° | 5.132 to 161.35 |
| Index ranges | -17 ≤ h ≤ 23, -13 ≤ k ≤ 13, -30 ≤ l ≤ 30 |
| Reflections collected | 75794 |
| Independent reflections | 9242 [R_int_ = 0.0506, R_sigma_ = 0.0295] |
| Data/restraints/parameters | 9242/0/601 |
| Goodness-of-fit on F^2^ | 1.066 |
| Final R indexes [I>=2σ (I)] | R_1_ = 0.0351, wR_2_ = 0.0858 |
| Final R indexes [all data] | R_1_ = 0.0408, wR_2_ = 0.0886 |
| Largest diff. peak/hole / e Å^-3^ | 0.81/-0.58 |

| Table S10: Interatomic distances for complex [Cu_2_(trop_3_P)(TFA)]BF_4_ (**5**).. | | | | | | |
| --- | --- | --- | --- | --- | --- | --- |
| **Atom** | **Atom** | **Length/Å** |  | **Atom** | **Atom** | **Length/Å** |
| Cu1 | Cu2 | 2.4573(4) |  | C16 | C30 | 1.520(3) |
| Cu1 | P1 | 2.5635(5) |  | C17 | C18 | 1.397(3) |
| Cu1 | O1 | 2.1685(15) |  | C17 | C22 | 1.406(3) |
| Cu1 | C8 | 2.2485(19) |  | C18 | C19 | 1.388(3) |
| Cu1 | C9 | 2.1358(18) |  | C19 | C20 | 1.391(4) |
| Cu1 | C23 | 2.1806(19) |  | C20 | C21 | 1.380(4) |
| Cu1 | C24 | 2.1578(19) |  | C21 | C22 | 1.408(3) |
| Cu2 | P1 | 2.2008(5) |  | C22 | C23 | 1.467(3) |
| Cu2 | O2 | 1.9540(16) |  | C23 | C24 | 1.373(3) |
| Cu2 | C38 | 2.234(2) |  | C24 | C25 | 1.464(3) |
| Cu2 | C39 | 2.265(2) |  | C25 | C26 | 1.406(3) |
| S1 | O1 | 1.4526(16) |  | C25 | C30 | 1.412(3) |
| S1 | O2 | 1.4727(16) |  | C26 | C27 | 1.380(3) |
| S1 | O3 | 1.4173(18) |  | C27 | C28 | 1.395(3) |
| S1 | C46 | 1.824(2) |  | C28 | C29 | 1.390(3) |
| P1 | C1 | 1.9030(19) |  | C29 | C30 | 1.394(3) |
| P1 | C16 | 1.9010(19) |  | C31 | C32 | 1.515(3) |
| P1 | C31 | 1.9251(19) |  | C31 | C45 | 1.511(3) |
| F1 | C46 | 1.329(3) |  | C32 | C33 | 1.400(3) |
| F2 | C46 | 1.322(3) |  | C32 | C37 | 1.409(3) |
| F3 | C46 | 1.327(3) |  | C33 | C34 | 1.396(3) |
| C1 | C2 | 1.507(3) |  | C34 | C35 | 1.378(4) |
| C1 | C15 | 1.522(3) |  | C35 | C36 | 1.375(4) |
| C2 | C3 | 1.391(3) |  | C36 | C37 | 1.407(3) |
| C2 | C7 | 1.404(3) |  | C37 | C38 | 1.465(3) |
| C3 | C4 | 1.386(3) |  | C38 | C39 | 1.367(3) |
| C4 | C5 | 1.387(3) |  | C39 | C40 | 1.465(3) |
| C5 | C6 | 1.387(3) |  | C40 | C41 | 1.405(3) |
| C6 | C7 | 1.406(3) |  | C40 | C45 | 1.407(3) |
| C7 | C8 | 1.467(3) |  | C41 | C42 | 1.384(3) |
| C8 | C9 | 1.371(3) |  | C42 | C43 | 1.386(3) |
| C9 | C10 | 1.463(3) |  | C43 | C44 | 1.387(3) |
| C10 | C11 | 1.408(3) |  | C44 | C45 | 1.397(3) |
| C10 | C15 | 1.410(3) |  | F4 | B1 | 1.403(3) |
| C11 | C12 | 1.384(3) |  | F5 | B1 | 1.387(3) |
| C12 | C13 | 1.388(3) |  | F6 | B1 | 1.390(3) |
| C13 | C14 | 1.385(3) |  | F7 | B1 | 1.387(3) |
| C14 | C15 | 1.395(3) |  | Cl1 | C47 | 1.763(3) |
| C16 | C17 | 1.522(3) |  | Cl2 | C47 | 1.750(3) |

Complex **6:**

Figure 9 Molecular structure of complex **6** with displacement ellipsoids drawn at 50 % probability level. H atoms and a BF_4_^-^ -anion are omitted for clarity. The component with minor occupancy (40 %) of the disordered CF_3_-group is shown in turquoise (C in black, P in pink, Cu in blue, O in red, F in green).

Refinement details: One CF3-group is strongly disordered over two positions and has been described using anisotropic displacement parameters. A displacement restraint (RIGU) with esds smaller than the default values has been used for the triflate ligand, while the displacement parameters of F1 and F1A were constraint to be equal (EADP). The six H-atoms at the olefinic C=C_trop_ binding site were located in the difference Fourier map and refined freely.

| Table S11: Crystallographic data for complex [Cu_2_(trop_3_P)(TFA)]BF_4_ (**6**). | |
| --- | --- |
| Identification code | dh_cu086_1_auto |
| Deposition number | CCDC-2501176 |
| Empirical formula | C_47_H_33_BCu_2_F_7_O_2_P |
| Formula weight | 931.59 |
| Temperature/K | 100.00(10) |
| Crystal system | monoclinic |
| Space group | *P*2_1_/*n* |
| a/Å | 10.17640(10) |
| b/Å | 19.1930(2) |
| c/Å | 19.2680(2) |
| α/° | 90 |
| β/° | 90.9750(10) |
| γ/° | 90 |
| Volume/Å^3^ | 3762.80(7) |
| Z | 4 |
| ρ_calc_g/cm^3^ | 1.644 |
| μ/mm^‑1^ | 2.467 |
| F(000) | 1888.0 |
| Crystal size/mm^3^ | 0.115 × 0.067 × 0.049 |
| Radiation | Cu Kα (λ = 1.54184) |
| 2Θ range for data collection/° | 6.5 to 160.656 |
| Index ranges | -12 ≤ h ≤ 13, -24 ≤ k ≤ 22, -24 ≤ l ≤ 22 |
| Reflections collected | 37485 |
| Independent reflections | 8066 [R_int_ = 0.0288, R_sigma_ = 0.0245] |
| Data/restraints/parameters | 8066/81/596 |
| Goodness-of-fit on F^2^ | 1.108 |
| Final R indexes [I>=2σ (I)] | R_1_ = 0.0359, wR_2_ = 0.0939 |
| Final R indexes [all data] | R_1_ = 0.0400, wR_2_ = 0.0960 |
| Largest diff. peak/hole / e Å^-3^ | 0.95/-0.43 |

| Table S12: Interatomic distances for complex [Cu_2_(trop_3_P)(TFA)]BF_4_ (**6**). | | | | | | |
| --- | --- | --- | --- | --- | --- | --- |
| **Atom** | **Atom** | **Length/Å** |  | **Atom** | **Atom** | **Length/Å** |
| Cu1 | Cu2 | 2.4320(4) |  | C21 | C22 | 1.402(3) |
| Cu1 | P1 | 2.2267(5) |  | C22 | C23 | 1.466(3) |
| Cu1 | O1 | 1.9552(14) |  | C23 | C24 | 1.367(3) |
| Cu1 | C8 | 2.2542(19) |  | C24 | C25 | 1.464(3) |
| Cu1 | C9 | 2.2744(19) |  | C25 | C26 | 1.404(3) |
| Cu2 | P1 | 2.5323(5) |  | C25 | C30 | 1.405(3) |
| Cu2 | O2 | 2.0747(15) |  | C26 | C27 | 1.379(4) |
| Cu2 | C23 | 2.159(2) |  | C27 | C28 | 1.374(4) |
| Cu2 | C24 | 2.227(2) |  | C28 | C29 | 1.392(4) |
| Cu2 | C38 | 2.182(2) |  | C29 | C30 | 1.391(3) |
| Cu2 | C39 | 2.188(2) |  | C31 | C32 | 1.514(3) |
| P1 | C1 | 1.9202(18) |  | C31 | C45 | 1.525(3) |
| P1 | C16 | 1.910(2) |  | C32 | C33 | 1.396(3) |
| P1 | C31 | 1.904(2) |  | C32 | C37 | 1.404(3) |
| O1 | C46 | 1.261(2) |  | C33 | C34 | 1.394(4) |
| O2 | C46 | 1.229(3) |  | C34 | C35 | 1.389(5) |
| C1 | C2 | 1.507(3) |  | C35 | C36 | 1.367(4) |
| C1 | C15 | 1.513(3) |  | C36 | C37 | 1.408(3) |
| C2 | C3 | 1.402(3) |  | C37 | C38 | 1.455(3) |
| C2 | C7 | 1.410(3) |  | C38 | C39 | 1.370(3) |
| C3 | C4 | 1.385(3) |  | C39 | C40 | 1.465(3) |
| C4 | C5 | 1.390(3) |  | C40 | C41 | 1.406(3) |
| C5 | C6 | 1.384(3) |  | C40 | C45 | 1.404(3) |
| C6 | C7 | 1.398(3) |  | C41 | C42 | 1.381(3) |
| C7 | C8 | 1.468(3) |  | C42 | C43 | 1.379(4) |
| C8 | C9 | 1.362(3) |  | C43 | C44 | 1.387(4) |
| C9 | C10 | 1.462(3) |  | C44 | C45 | 1.396(3) |
| C10 | C11 | 1.408(3) |  | C46 | C47 | 1.509(10) |
| C10 | C15 | 1.409(3) |  | C46 | C47A | 1.602(15) |
| C11 | C12 | 1.384(3) |  | F1 | C47 | 1.275(19) |
| C12 | C13 | 1.383(3) |  | F2 | C47 | 1.247(16) |
| C13 | C14 | 1.387(3) |  | F3 | C47 | 1.524(17) |
| C14 | C15 | 1.397(3) |  | F1A | C47A | 1.42(3) |
| C16 | C17 | 1.517(3) |  | F2A | C47A | 1.53(3) |
| C16 | C30 | 1.513(3) |  | F3A | C47A | 1.08(2) |
| C17 | C18 | 1.396(3) |  | F4 | B1 | 1.403(3) |
| C17 | C22 | 1.400(3) |  | F5 | B1 | 1.365(3) |
| C18 | C19 | 1.382(4) |  | F6 | B1 | 1.400(3) |
| C19 | C20 | 1.373(5) |  | F7 | B1 | 1.365(3) |
| C20 | C21 | 1.396(4) |  |  |  |  |

Table S13. Cu-C distances in complexes **2**, **3**, **4**, **4’**, **5** and **6**.

| Complex | Central atom | Centroid (ct) | Distance /Å | Atom1=Atom2 | Distance Cu-Atom1 /Å | Distance Cu-Atom2  /Å |
| --- | --- | --- | --- | --- | --- | --- |
| **2** | Cu1 | 1 | 2.981(2) | C8=C9 | 2.866(3) | 3.233(3) |
|  |  | 2 | 2.689(2) | C23=C24 | 2.672(3) | 2.870(3) |
|  |  | 3 | 3.390(2) | C38=C39 | 3.074(3) | 3.799(3) |
| **3** | Cu1 | 1 | 2.875(7) | C8=C9 | 3.12(1) | 2.776(9) |
|  |  | 2 | 2.505(6) | C23=C24 | 2.695(9) | 2.495(8) |
|  |  | 3 | 2.832(6) | C38=C39 | 3.092(9) | 2.716(8) |
|  | Cu2 | 1’ | 3.001(6) | C53=C54 | 2.857(9) | 3.283(8) |
|  |  | 2’ | 2.502(5) | C68=C69 | 2.475(8) | 2.669(7) |
|  |  | 3’ | 2.786(6) | C83=C84 | 2.702(8) | 3.018(8) |
| **4** | Cu1 | 1 | 2.070(5) | C8=C9 | 2.181(8) | 2.180(7) |
|  | Cu2 | 2 | 2.068(5) | C23=C24 | 2.170(7) | 2.183(8) |
|  |  | 3 | 2.050(5) | C38=C39 | 2.189(8) | 2.133(7) |
| **4’** | Cu1 | 1 | 2.222(3) | C8=C9 | 2.262(4) | 2.384(4) |
|  |  | 2 | 2.317(3) | C23=C24 | 2.322(4) | 2.502(4) |
|  |  | 3 | 3.422(3) | C38=C39 | 3.341(4) | 3.629(4) |
|  | Cu2 | 1’ | 2.182(3) | C53=C54 | 2.260(4) | 2.306(5) |
|  |  | 2’ | 2.182(3) | C53=C54 | 2.260(4) | 2.306(5) |
|  |  | 3’ | 3.464(3) | C68=C68 | 3.527(4) | 3.527(4) |
| **5** | Cu1 | 1 | 2.0830(17) | C8=C9 | 2.249(2) | 2.136(2) |
|  |  | 2 | 2.0578(14) | C23=C24 | 2.181(2) | 2.1578(18) |
|  | Cu2 | 3 | 2.1433(15) | C38=C39 | 2.234(2) | 2.265(2) |
| **6** | Cu1 | 1 | 2.1596(14) | C8=C9 | 2.2542(19) | 2.2745(19) |
|  | Cu2 | 2 | 2.0843(16) | C23=C24 | 2.159(2) | 2.227(2) |
|  |  | 3 | 2.0748(16) | C38=C39 | 2.182(2) | 2.188(2) |

# 7 References

[1]	G. Berti, *J. Org. Chem.* **1957**, *22*, 230–230.

[2]	J. Oswald, C. Fekete, M. Baker, M. Bispinghoff, P.G. Pringle, N. Weferling, Z. Benkő, H. Grützmacher, *Angew. Chem. Int. Ed.* **2025**, e202501656.

[3] G.M. Sheldrick, *Acta Cryst.* **2015**, A71, 3-8.

[4] G.M. Sheldrick, *Acta Cryst.* **2015**, C71, 3-8.

[5]	O. V. Dolomanov, L. J. Bourhis, R. J. Gildea, J. a. K. Howard, H. Puschmann, *J Appl Cryst* **2009**, *42*, 339–341.

[6]	U. Fischbach, H. Rüegger, H. Grützmacher, *European Journal of Inorganic Chemistry* **2007**, *2007*, 2654–2667.
